# Spike-Based Bayesian-Hebbian Learning of Temporal Sequences

**DOI:** 10.1371/journal.pcbi.1004954

**Published:** 2016-05-23

**Authors:** Philip J. Tully, Henrik Lindén, Matthias H. Hennig, Anders Lansner

**Affiliations:** 1 Department of Computational Science and Technology, Royal Institute of Technology (KTH), Stockholm, Sweden; 2 Stockholm Brain Institute, Karolinska Institute, Stockholm, Sweden; 3 Institute for Adaptive and Neural Computation, School of Informatics, University of Edinburgh, Edinburgh, United Kingdom; 4 Department of Neuroscience and Pharmacology, Faculty of Health and Medical Sciences, University of Copenhagen, Copenhagen, Denmark; 5 Department of Numerical Analysis and Computer Science, Stockholm University, Stockholm, Sweden; Research Center Jülich, GERMANY

## Abstract

Many cognitive and motor functions are enabled by the temporal representation and processing of stimuli, but it remains an open issue how neocortical microcircuits can reliably encode and replay such sequences of information. To better understand this, a modular attractor memory network is proposed in which meta-stable sequential attractor transitions are learned through changes to synaptic weights and intrinsic excitabilities via the spike-based Bayesian Confidence Propagation Neural Network (BCPNN) learning rule. We find that the formation of distributed memories, embodied by increased periods of firing in pools of excitatory neurons, together with asymmetrical associations between these distinct network states, can be acquired through plasticity. The model’s feasibility is demonstrated using simulations of adaptive exponential integrate-and-fire model neurons (AdEx). We show that the learning and speed of sequence replay depends on a confluence of biophysically relevant parameters including stimulus duration, level of background noise, ratio of synaptic currents, and strengths of short-term depression and adaptation. Moreover, sequence elements are shown to flexibly participate multiple times in the sequence, suggesting that spiking attractor networks of this type can support an efficient combinatorial code. The model provides a principled approach towards understanding how multiple interacting plasticity mechanisms can coordinate hetero-associative learning in unison.

## Author Summary

From one moment to the next, in an ever-changing world, and awash in a deluge of sensory data, the brain fluidly guides our actions throughout an astonishing variety of tasks. Processing this ongoing bombardment of information is a fundamental problem faced by its underlying neural circuits. Given that the structure of our actions along with the organization of the environment in which they are performed can be intuitively decomposed into sequences of simpler patterns, an encoding strategy reflecting the temporal nature of these patterns should offer an efficient approach for assembling more complex memories and behaviors. We present a model that demonstrates how activity could propagate through recurrent cortical microcircuits as a result of a learning rule based on neurobiologically plausible time courses and dynamics. The model predicts that the interaction between several learning and dynamical processes constitute a compound mnemonic engram that can flexibly generate sequential step-wise increases of activity within neural populations.

## Introduction

Converging threads of evidence suggest that neural ensemble dynamics consist of intermittent population bursts with abrupt sequential transitions occurring on the order of hundreds of milliseconds. Indicating conserved underlying mechanisms, this hallmark of activity occurs in a diverse array of behaviors including during motor [1–3], sensory [4,5], memory [6–9], and decision making [10,11] tasks. Similar population activity regimes are observed in cortical reactivations during sleep following motor performance or sensory exposure while the animal is awake [12–14]. These patterns routinely arise in the form of transient neuronal coalitions that experience concomitant shifts in their firing rates and manifest as stereotypical trajectories taken through the state space of neocortical networks.

Such discrete states of increased activity within which firing rates are approximately stationary allude to the attractor memory paradigm, which has been elaborated theoretically [15,16] and biophysically [17] from Hebb’s early notion of cell assemblies [18]. Empirical evidence for attractor-like dynamics notwithstanding [19,20], reconciling attractor network models with the irregularity of cortical spiking observed *in vivo* has been problematic [21]. A proposed model circumvents this issue by introducing modularity in the form of soft winner-take-all (WTA) hypercolumns, which bestow irregular firing regimes and allow for switching between a low-rate background state and elevated attractor activations [22]. However, spike-based procedures for learning the connectivity required to support attractor dynamics in this model have not yet been considered, and temporal associations between different attractor states remain absent.

More generally, it remains an open question how stable sequential dynamics could self-organize within recurrent networks in a biologically meaningful way [23,24]. Despite issues regarding its ubiquity [25] and fidelity [26] as a generic learning rule, spike-timing dependent plasticity (STDP) [27] has often been exploited for sequence learning. Temporal causality is presumably beneficial for feed-forward structural development, but complementary mechanisms are typically required due to the inherent instability of phenomenological STDP rules [28].

For reliable temporal sequence production, STDP has been coupled with a slew of biologically-inspired phenomena including network-wide neuromodulatory interactions [29], synaptic weight normalization [30–32] and intrinsic excitability [33,34]. Although such modeling studies may employ the fewest number of mechanisms for the sake of computational simplicity, they implicitly ignore the combination of these mechanisms among potentially many other interacting dynamical processes.

How can one begin to understand these complicated synergies in a principled way? We propose that an attractor memory network could learn temporal sequences using the spike-based BCPNN learning rule [35]. Although the approach encompasses functionally diverse mechanisms including Hebbian, homeostatic, neuromodulated, and intrinsic plasticity, it can be straightforwardly understood since it is neatly encapsulated within the framework of probabilistic inference [36,37].

To enable the collective cortical transitions of the sort aforementioned, synaptic plasticity occurs on separate short and long time scales. Fast AMPA channels stabilize within-attractor activity, whereas temporal sequence development hinges upon slower NMDA channels whose delayed activations govern transitions between attractors [38,39]. The learning rule interprets these parameters as establishing a window of temporal integration for synaptic plasticity.

Through AdEx neuron simulations, the model can account for several noteworthy aspects of temporal sequence development including dependence on trained temporal interval [40], performance of prospective or retrospective replay regardless of the temporal training direction [41,42], cue-triggered recall [43], and co-temporal neural representation of serial order [1]. It provides a basis to explore mechanisms by which sequential cortical activity is compressed in time [12,13,43], and demonstrates that relative replay speed is determined by a combination of intrinsic network parameters as well as the speed of conditioning stimuli. Lastly, we illustrate the flexibility of the learning rule in the network using experimentally motivated stimulus configurations, and find that it is able to complete and disambiguate overlapping patterns [44] through successful retrieval of sequential attractor trajectories.

## Results

### A Spike-Based BCPNN Learning Model

We implemented a computational model of a cortical microcircuit inspired by previous work [45] that was topographically structured according to the columnar organization of neocortex [46,47]. The network consisted of 2700 pyramidal cells and 270 basket cells divided into nine hypercolumns (*N*_*HC*_ = 9), each consisting of 300 pyramidal cells and 30 basket cells. Pyramidal cells had spike-frequency adaptation according to the AdEx model (See Eq 6 in Methods).

**Fig 1:**
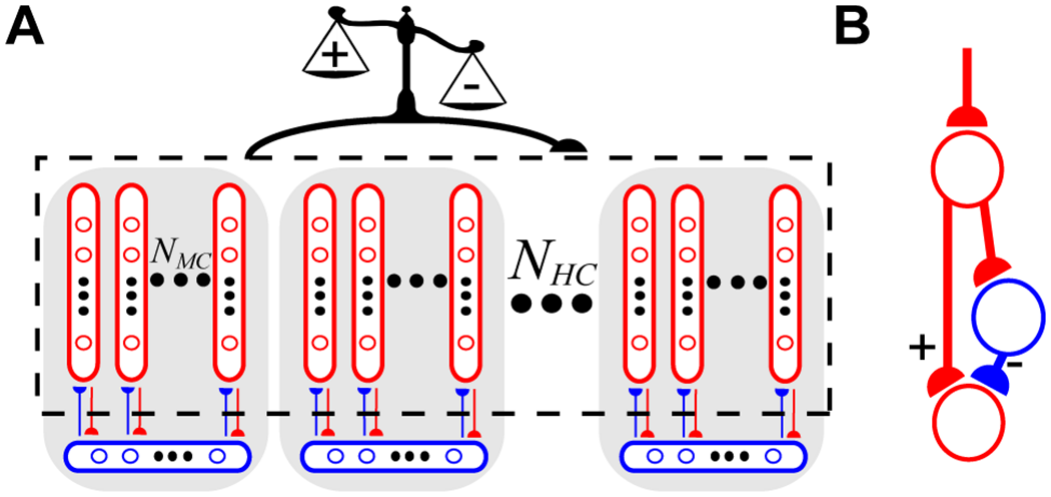
Organization of a neocortical microcircuit. **(A)** The WTA hypercolumns (shaded gray areas) consisted of excitatory neurons (red circles) belonging to minicolumns (red ovals) that innervated (red arrows) local populations (blue ovals) of basket cells (blue circles), which provided inhibitory feedback (blue arrows). Nonspecific BCPNN synapses (black arrow) were recurrently connected amongst all excitatory neurons (black dotted box), with learned weights (scale) determined by pre- and postsynaptic spike activity. Semicircular arrowheads indicate the postsynaptic target. **(B)** Schematic of a polysynaptic subcircuit that elicited a net excitatory effect on the postsynaptic neuron when the BCPNN weight was positive (**+** pathway), while a negative BCPNN weight can be thought of as being converted to inhibition via excitation of a local inhibitory interneuron (− pathway).

The pyramidal cells in each hypercolumn were further divided into ten minicolumns (*N*_*MC*_ = 10), each consisting of 30 pyramidal cells [48]. The pyramidal cells in each minicolumn laterally projected to and received feedback from the 30 local inhibitory basket cells within their own hypercolumn to form competitive WTA subnetworks [49]. These connections were static and consisted of both excitatory AMPA-mediated pyramidal-to-basket and inhibitory GABAergic basket-to-pyramidal connections with connection probabilities set to 0.7. In addition to the static local (i.e., within-hypercolumn) feedback connections, there were plastic BCPNN synapses (see below), which randomly formed AMPA- and NMDA-mediated connections among all pyramidal cells across the network with distant-dependent axonal delays (see Eq 5 in Methods) and a connection probability of 0.25 (Fig 1A). These connections also exhibited short-term depression (see Eq 8 in Methods).

The strengths of AMPA and NMDA synapses between pyramidal cells along with the intrinsic excitabilities of all pyramidal cells underwent plasticity according to the spike-based BCPNN learning rule. BCPNN makes use of local traces that estimate the firing rates of pre- and postsynaptic neurons along with their coactivations, respectively referred to below as *P*_*i*_, *P*_*j*_, and *P*_*ij*_. Statistics of neural input and output activity are used to infer a synaptic weight *w*_*ij*_ between pre- and postsynaptic neurons *i* and *j*, and an intrinsic excitability *β*_*j*_ of postsynaptic neuron *j*. For simplicity, the separate synaptic traces in Eqs 1–3 are specified by either pre- (*i*), postsynaptic (*j*), or both (*ij*) neural variables depending on their relative source(s) of activity [35].

Pre- *S*_*i*_ and postsynaptic *S*_*j*_ spike trains are formally described by summed Dirac delta pulses *δ*(·) with respective spike times $t_{sp}^{i}$ and $t_{sp}^{j}$:

$$S_{i}(t)=\sum_{sp}\delta (t-t_{sp}^{i}),\;S_{j}(t)=\sum_{sp}\delta (t-t_{sp}^{j}).$$ (1)

Smoothing was performed using a system of two coupled exponentially weighted moving averages. The first level of traces, the *Z* traces, lowpass filtered *S*_*i*_ and *S*_*j*_:

$$\tau_{z_{i}}^{syn}\frac{dZ_{i}}{dt}=\frac{S_{i}}{f_{max}\Delta t}-Z_{i}+\varepsilon ,\;\tau_{z_{j}}^{syn}\frac{dZ_{j}}{dt}=\frac{S_{j}}{f_{\text{max}}\Delta t}-Z_{j}+\varepsilon .$$ (2)

Setting *f*_*max*_ = 20 Hz established a normalizing linear transformation between neuronal spike rate ∈ [*f*_*min*_, *f*_*max*_] and probability space ∈ [*ε*, 1] (see Eqs 5–7 in Methods) such that the average value of the *Z* traces over time was approximately 1 when a neuron fired at *f*_*max*_, *ε* = 0.01 represented the lowest attainable probability estimate, *f*_*min*_ = *εf*_*max*_ = 0.2 Hz the corresponding lowest firing rate, Δ*t* = 1 ms denoted the spike duration, and *syn* represented AMPA or NMDA synapses. Presynaptic *Z* trace time constants were set to $\tau_{z_{i}}^{AMPA}$ = 5 ms and $\tau_{z_{i}}^{NMDA}$ = 150 ms, the latter reflected the slow closing dynamics of NMDA receptor gated channels. Postsynaptic *Z* trace time constants $\tau_{z_{j}}^{AMPA}$ = $\tau_{z_{j}}^{NMDA}$ = 5 ms reflected fast closing dynamics of AMPA receptor gated channels and membrane depolarization due to spike back-propagation, respectively [50].

Activation and coactivation probabilities were estimated by filtering the *Z* traces:

$$\tau_{p}\frac{dP_{i}}{dt}=\kappa (Z_{i}-P_{i}),\;\tau_{p}\frac{dP_{j}}{dt}=\kappa (Z_{j}-P_{j}),\;\tau_{p}\frac{dP_{ij}}{dt}=\kappa (Z_{i}Z_{j}-P_{ij}).$$ (3)

AMPA channel phosphorylation and mobilization from reserve pools, gene expression and protein synthesis are potential candidates for mediating Hebbian synaptic plasticity and memory formation in a manner prescribed by the *P* traces [51,52]. These traces constitute the memory itself, which decays in palimpsest fashion.

The time constant for the *P* traces, *τ*_*p*_, reflected the extended time course of the downstream processes. We simply imposed $\tau_{z_{i}}^{syn}$, $\tau_{z_{j}}^{syn}$ < *τ*_*p*_, whereas in reality the timescale of *τ*_*p*_ can exhibit high variability ranging from seconds to months [53]. Since the time it took to learn increased proportionally to *τ*_*p*_, it was set to 5000 ms to speed up simulations while preserving the dynamics studied. *P* traces had the same time constants because they reflected estimated spike rates whose updates incrementally converged to approximate probabilities (see Eq 1–3 in S1 Appendix).

*P* traces were modulated by a globally applied ‘print-now’ factor *κ* ∈ [0, 1], which signaled the relevance of recent synaptic events in the spirit of what would be expected from a neuromodulator like dopamine [54]. By assuming that neurons were minimally active, the *P* traces were always positive, allowing their logarithmic transformation (Eq 4, see below) to remain defined.

The full derivation starting from Bayes’ rule is delineated elsewhere [35] and summarized in S1 Appendix. Probabilistic computations on the network level can be approximated by individual neuronal biases $I_{\beta_{j}}$ and synaptic weights $w_{ij}^{syn}$:

$$I_{\beta_{j}}=\beta_{gain}\text{log}(P_{j}),\;w_{ij}^{syn}=w_{gain}^{syn}\text{log}\frac{P_{ij}}{P_{i}P_{j}}.$$ (4)

The dimensionless *β*_*j*_ = log(*P*_*j*_) component quantified a general level of excitability and spiking of the postsynaptic neuron as it was solely dependent upon that neuron’s relative activity [55]. *β*_*j*_ was scaled by *β*_*gain*_ = 50 pA to give $I_{\beta_{j}}$, an activity-dependent intrinsic membrane current of an AdEx model neuron (see Eq 6 in Methods) like the A-type K+ channel [56] or TRP channel [57]. The strength of a BCPNN synapse was $w_{ij}^{syn}$. The peak amplitude of the conductance transient was determined by the logarithmic BCPNN weight multiplied with $w_{gain}^{syn}$ using values $w_{gain}^{AMPA}$ = 6.02 nS and $w_{gain}^{NMDA}$ = 1.22 nS. Their ratio was the AMPA/NMDA ratio, which was tested at different levels due to variations in reported values [58].

It was assumed that stimulus arrival coincided with an increase in *κ*, the third factor of the learning rule, for all BCPNN synapses and pyramidal cells. Mimicking dopamine activation responses as learning progresses for novel stimuli [59,60], *κ* temporarily permitted changes in all $w_{ij}^{syn}$ and $I_{\beta_{j}}$ through global modulation of *τ*_*p*_ (Eq 3). During interleaved quiescent periods when no stimulus was deemed relevant, *κ* was clamped to 0. In practice *κ* = 0 disabled learning by freezing $w_{ij}^{syn}$ and $I_{\beta_{j}}$, allowing for post-training simulation in off-line mode [61]. During training periods, *κ* = 1 while both *β*_*gain*_ and $w_{gain}^{syn}$ were set to 0, and during recall periods, *κ* = 0 while both *β*_*gain*_ and $w_{gain}^{syn}$ were positive.

The logarithmic transformation from Eq 4 was motivated by the Bayesian underpinnings of the learning rule, but this caveat meant that $w_{ij}^{syn}$ multiplexed both positive and negative signals. Learning of excitatory or inhibitory weights was interpreted in the context of a monosynaptic excitatory connection with conductance set by the positive component of $w_{ij}^{syn}$ alongside a disynaptic inhibitory connection [62–65] set by the negative component (Fig 1B). In practice, when the sign of the BCPNN weight turned negative, an inhibitory reversal potential was used instead of an excitatory one (see Eq 7 in Methods). The rationale for the development of negative weights is expounded upon in the Discussion.

**Fig 2:**
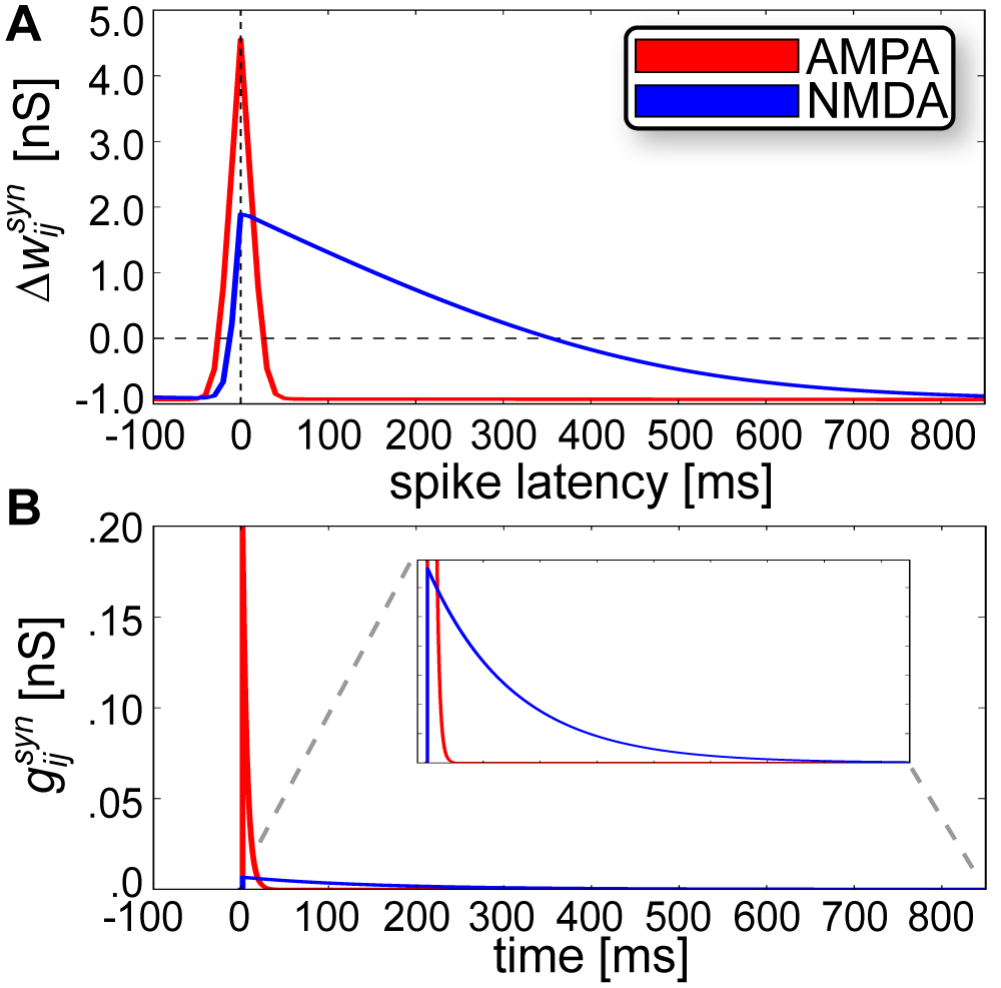
Matching learning and synaptic time constants for AMPA and NMDA. **(A)** Absolute change in peak synaptic amplitude after 100 pre- and postsynaptic events at 1 Hz, where spike latency > 0 denotes a pre before post pairing. **(B)** Postsynaptic conductances elicited by EPSPs via AMPA and NMDA receptors display similar time courses of activation to their corresponding learning window widths from (A). The inset provides a zoomed view of the area contained within the dotted gray lines.

Following previous theoretical studies [66–68] the shape of the learning kernel (Fig 2A) was linked to the shape of the EPSP measured by change in synaptic conductance $g_{ij}^{syn}$ (see Eq 9 in Methods), which was determined by synaptic time constants *τ*_*AMPA*_ = 5 ms and *τ*_*NMDA*_ = 150 ms [69] (Fig 2B). We set $\tau_{z_{i}}^{NMDA}$ = *τ*_*NMDA*_ and $\tau_{z_{i}}^{AMPA}$ = *τ*_*AMPA*_, entailing that the NMDA component was subject to plasticity [70] and resulting in a temporally shifted window function [71].

### Learning Sequential Attractors Using Spike-Based BCPNN

**Fig 3:**
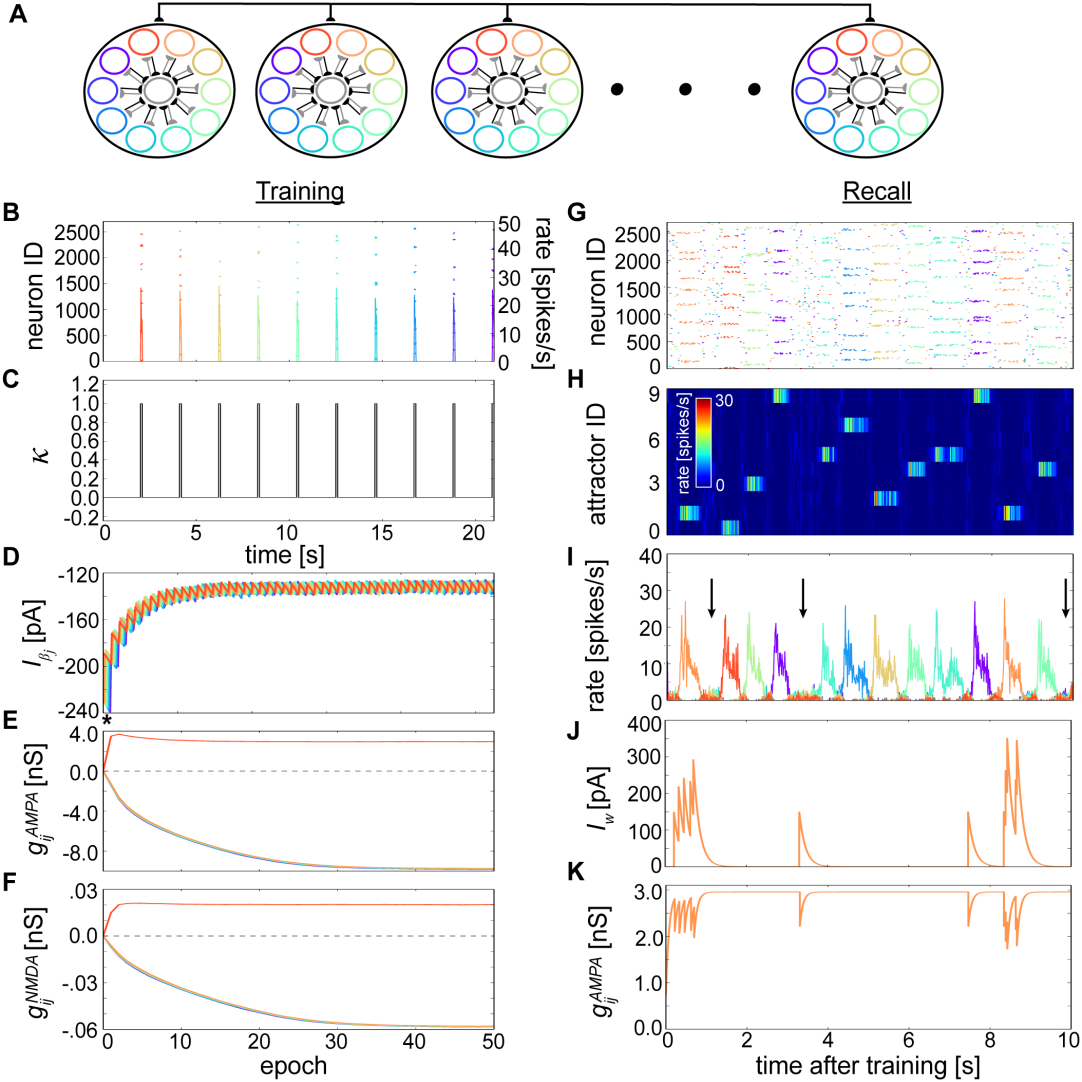
Learning spontaneously wandering attractor states. **(A)** Alternate network schematic with hypercolumns (large black circles), along with their member minicolumns (smaller colored circles) and local basket cell populations (centered gray circles). Minicolumns with the same color across different hypercolumns belong to the same pattern; these color indicators are used in the following subfigures. **(B)** Rastergram (10x downsampled, henceforth) and superimposed firing rates (10 ms bins averaged per pattern, henceforth) associated with the first training epoch. **(C)** Progression of the ‘print-now’ *κ* signal whose brief activations are synchronized with the incoming stimuli from (B). **(D)** Development of $I_{\beta_{j}}$ during training and averaged over 50 randomly selected neurons per pattern. The period of time shown in (B, C) can be discerned as * for reference. **(E)** Development of average $g_{ij}^{AMPA}$ during training that project from neurons belonging to the first stimulated (i.e. red) pattern, with colors denoting target postsynaptic neuron pattern. **(F)** Same as (E), except showing $g_{ij}^{NMDA}$ development during training. **(G)** Rastergram snapshot of excitatory neurons during recall. **(H)** Relative average firing rates based on (G) and sorted by attractor membership. Each row represents the firing rate of one attractor. **(I)** Average firing rate of the attractors displaying the random progression of the network through state space. Arrows highlight the ground state, which are competitive phases lacking a dominantly active attractor. **(J)** Evolution of the adaptation current *I*_*w*_ for a single neuron whose activity builds up and attenuates as it enters and exits attractor states. **(K)** Evolution of the same neuron’s dynamic AMPA strength due to short-term depression, whose postsynaptic target resides within the same attractor.

The network was initially trained using 10 orthogonal component patterns. Each pattern consisted of one active minicolumn per hypercolumn without overlap (Fig 3A), and was presented for *t*_*stim*_ = 100 ms using an inter-pulse interval (IPI) of 2 seconds (Fig 3B) during which *κ* was clamped to 0 (Fig 3C). Over the course of training, plastic changes transpired for intrinsic currents $I_{\beta_{j}}$ (Fig 3D) and weights $g_{ij}^{syn}$ in both AMPA (Fig 3E) and NMDA (Fig 3F) synapses. Neurons sharing patterns developed positive connections among themselves, but those belonging to different patterns developed strong negative ones. Minicolumn neurons belonging to the same hypercolumn had similar average connection strength profiles, so Fig 3E and 3F would look the same for a single minicolumn within a hypercolumn as they do here for neurons belonging to a single pattern across all hypercolumns. Learned quantities did not converge precisely but rather fluctuated within an interval. The amplitude of these fluctuations was determined by *τ*_*p*_ (S1 Fig). Similar firing rate statistics during training together with a vanishingly small *t*_*stim*_ in the limit of *τ*_*p*_ ensured approximately equal values for positive $g_{ij}^{syn}$ within patterns, for negative *$g_{ij}^{syn}$* between patterns, and for all $I_{\beta_{j}}$.

During memory recall, the network spontaneously recalled stored temporal sequences in the form of temporally coactive patterns (Fig 3G). Sorting the rastergram by pattern revealed clear ~500 ms periods of elevated firing rates shared by groups of neurons from different hypercolumns (Fig 3H). We refer to these periods as attractor states (see Methods). Network dynamics wandered from attractor to attractor, alternating in a seemingly random fashion, while neurons outside the currently active attractor were silenced due to lateral inhibition (Fig 3I). A non-coding ground state spontaneously emerged where individual active states were transiently interrupted by lower rate (~2 Hz) volatile periods in which neurons competed via WTA, thwarting any obvious selection of a winner. Attractor demise was precipitated by a combination of inhibition from the network together with neural and synaptic fatigue, which consisted of an enhanced spike-triggered adaptation current *I*_*w*_ (Fig 3J, see Eq 6 in Methods) and short-term depression that depleted available synaptic resources (Fig 3K, Eq 8) amid active attractor states.

**Fig 4:**
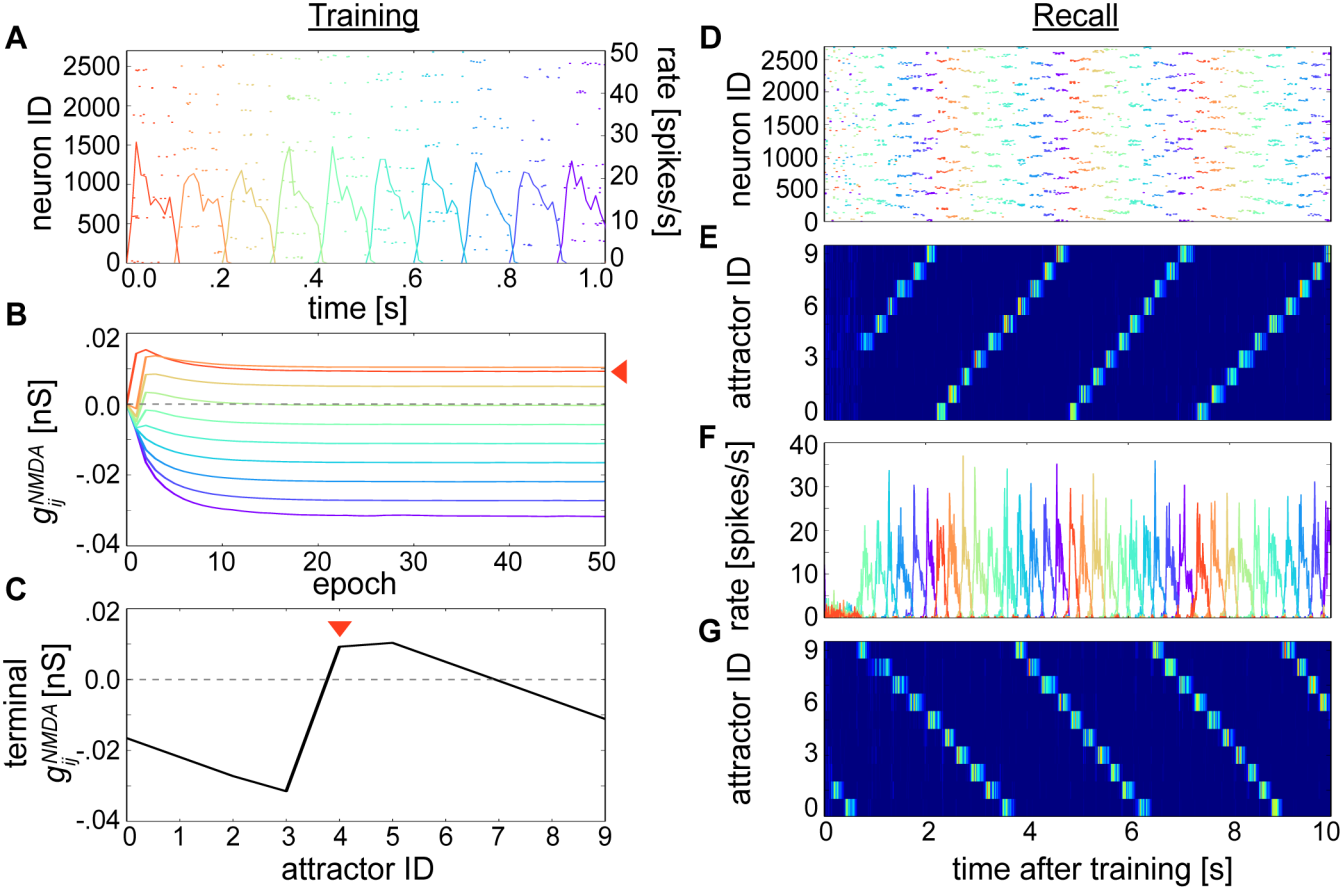
Learning sequential attractor states. **(A)** Rastergram and firing rates associated with the first out of 50 epochs of training. **(B)** Average $g_{ij}^{NMDA}$ during training emanating from neurons belonging to the first stimulated pattern as in Fig 3E (indicated with red arrow, colors denote target postsynaptic neuron pattern). Contrast the weight trajectories between patterns with Fig 3F. **(C)** Average $g_{ij}^{NMDA}$ after training that depicts an asymmetrical terminal weight profile. As in (B), the red arrow indicates the presynaptic perspective taken from the first stimulated pattern, which is aligned here at index 4. **(D)** Rastergram snapshot of excitatory neurons during recall. **(E)** Relative average firing rates based on (D) and sorted by attractor membership as in Fig 3H. The sequence is chronologically ordered according to the trained patterns from (A). **(F)** Average firing rate of attractors displaying the sequential progression of the network through state space. **(G)** Resulting recall after training the network by exchanging $\tau_{z_{i}}^{NMDA}$ for $\tau_{z_{j}}^{NMDA}$ showing the reverse traversal of attractor states from (D-F). Firing rates here and in (E) are coded according to the colorbar from Fig 3H.

A longer IPI (2 s) relative to the NMDA plasticity window width $\tau_{z_{i}}^{NMDA}$ (150 ms) meant that associations between different patterns did not develop. To demonstrate how BCPNN learning could memorize sequences, we altered the training protocol by decreasing IPIs and then reexamined network recall dynamics. We began by reducing the IPI to 0 ms (Fig 4A). Salient stimuli were always being encountered by the network in this case, so *κ* = 1 throughout. This enabled an evolution of $I_{\beta_{j}}$ as in Fig 3D and $g_{ij}^{AMPA}$ trajectories as in Fig 3E, except for the two neighboring patterns whose slightly higher $g_{ij}^{AMPA}$ arose from short temporal overlaps with $\tau_{z_{i}}^{AMPA}$ and $\tau_{z_{j}}^{AMPA}$ (S2A Fig). The otherwise similar learning outcomes can be appreciated by the fact that neurons were exposed to identical stimuli while *κ* = 1. Stimulus gaps previously encountered while *κ* = 0 (Fig 3A), which would have led to $g_{ij}^{syn}$ and $I_{\beta_{j}}$ decay if *κ* was nonzero, were essentially never learned.

However, the lower IPI did lead to a dispersion of higher average NMDA connection strengths that deviated markedly from Fig 3F (Fig 4B). Filtering by the BCPNN traces reflected relative stimulus presentation times, which created a discrepancy in the amount of overlap in the underlying running averages of coactivity traces *P*_*ij*_ (Eq 3, S2B Fig) when the IPI was reduced. Reflecting the temporal disparity between *t*_*stim*_ versus $\tau_{z_{i}}^{NMDA}$, the modified training procedure allowed the network to learn stronger feedforward associations than recurrent ones and produced an asymmetrical terminal $g_{ij}^{NMDA}$ structure (Fig 4C). NMDA synapses connecting excitatory cells in subsequently presented patterns formed progressively decreasing forward associations. Taken from the other perspective, trailing inhibition was learned in the form of negative-valued *$g_{ij}^{NMDA}$* towards antecedent pattern cells.

The resulting weight configuration reorganized the network dynamics, generating reliable sequential transitioning between attractor states (Fig 4D–4F). Sequential state traversal stably and perpetually repeated, though including longer temporal gaps between entire epochs rather than individual patterns could alleviate such cyclic behavior. Given the same training protocol as depicted in Fig 4A, switching the learning window widths $\tau_{z_{i}}^{NMDA}$ and $\tau_{z_{j}}^{NMDA}$ generated a mirror image of the *$g_{ij}^{NMDA}$* profile from Fig 4C (S2C Fig) and led to reverse recall of the stored patterns (Fig 4G).

**Fig 5:**
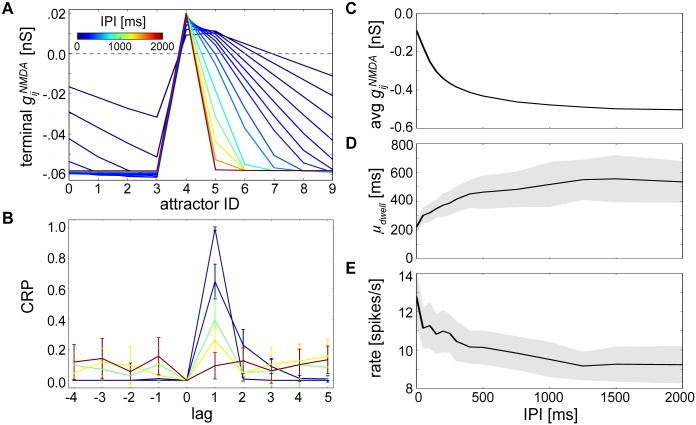
The temporal structure of neural recall dynamics reflects the temporal interval used during training. **(A)** Average $g_{ij}^{NMDA}$ after training as in Fig 4C (reproduced here by the 0 ms line) except now depicting terminal weight profiles for many differently trained networks with IPIs varying between 0 and 2000 ms. **(B)** CRP curves calculated for networks with representative IPIs = 0, 500, 1000, 1500 and 2000 ms after 1 minute of recall, with colors corresponding to (A). Increasing IPIs flattened the CRP curve, promoting attractor transition distribution evenness. Error bars reflect standard deviations. **(C)** Average strength of $g_{ij}^{NMDA}$ taken across entire networks after training for different IPIs, where the number of NMDA synapses in these separate networks was constant. **(D)** Average dwell times *μ*_*dwell*_ measured during 1 minute recall periods for entire networks trained with different IPIs. Shaded areas denote standard deviations here and in (E). **(E)** Average neural firing rates for attractors with dwell times corresponding to those measured in (D).

In networks trained using longer IPIs, the probability of transitioning from one attractor to another seemed uniform (Fig 3G–3I). However, attractor transitions became more deterministic as IPIs were decreased to 0 ms (Fig 4D–4F), thus there were differential effects of the training interval on neural recall dynamics [40]. Since the difference in recall dynamics between the two setups presented here could be mostly ascribed to the makeup of $g_{ij}^{NMDA}$ between neurons from different patterns (e.g. rather than $g_{ij}^{AMPA}$ or $I_{\beta_{j}}$), we expressed the learned model networks at different temporal intervals using their terminal $g_{ij}^{NMDA}$ profiles (Fig 5A).

To characterize the transformation from random to nonrandom temporal sequences depending on these resulting weight profiles, we measured the distribution of attractor transitions for different IPIs using CRP curves (see Methods). Shortening the IPI led to narrower CRP curves that peaked at lag 1 (Fig 5B), indicating this manipulation decreased recall heterogeneity and made forward transitions more likely. Networks trained with longer IPIs instead displayed CRP curves that hovered around chance levels.

A side effect of the learning procedure that led to varying degrees of asymmetry as a function of the IPI was the difference between total learned NMDA synaptic strengths averaged over each entire network (Fig 5C). In networks trained with shorter IPIs, surplus aggregate $g_{ij}^{NMDA}$, specifically $g_{ij}^{NMDA}$ projecting towards subsequent patterns, influenced not only the order of recall but also the resulting average dwell times *μ*_*dwell*_ (Fig 5D, see Methods) and firing rates (Fig 5E). A more pronounced effect of NMDA in low-IPI trained networks meant higher within-attractor firing rates that translated into stronger activation of adaptation and short-term depression, and therefore quicker initiation and cessation of individual attractors. Reduced NMDA in high-IPI trained networks meant that AMPA receptors could prolong attractor states further before succumbing to the pressures of neural and synaptic fatigue.

Specifically, lag 1 transitions (Fig 5B) conveyed the temporal extent of sequence replay. To ascertain whether the transformation in recall order dynamics from random to sequential was gradual or abrupt, we ran the same analysis but considered only the lag 1 transitions of the CRP curves (S3 Fig). We found that the temporal extent of sequence recall was limited with CRP curves reaching chance levels around IPI = 1500 ms [12]. The rather gradual decline in the CRP from the point at which forward transitions were inevitable, to the point at which their probability was indistinguishable from all other possible transitions in the network, demonstrates the capability of NMDA to influence the trajectory taken through state space depending upon trained temporal interval.

### Characterizing the Dynamics Underlying Temporal Sequence Recall

**Fig 6:**
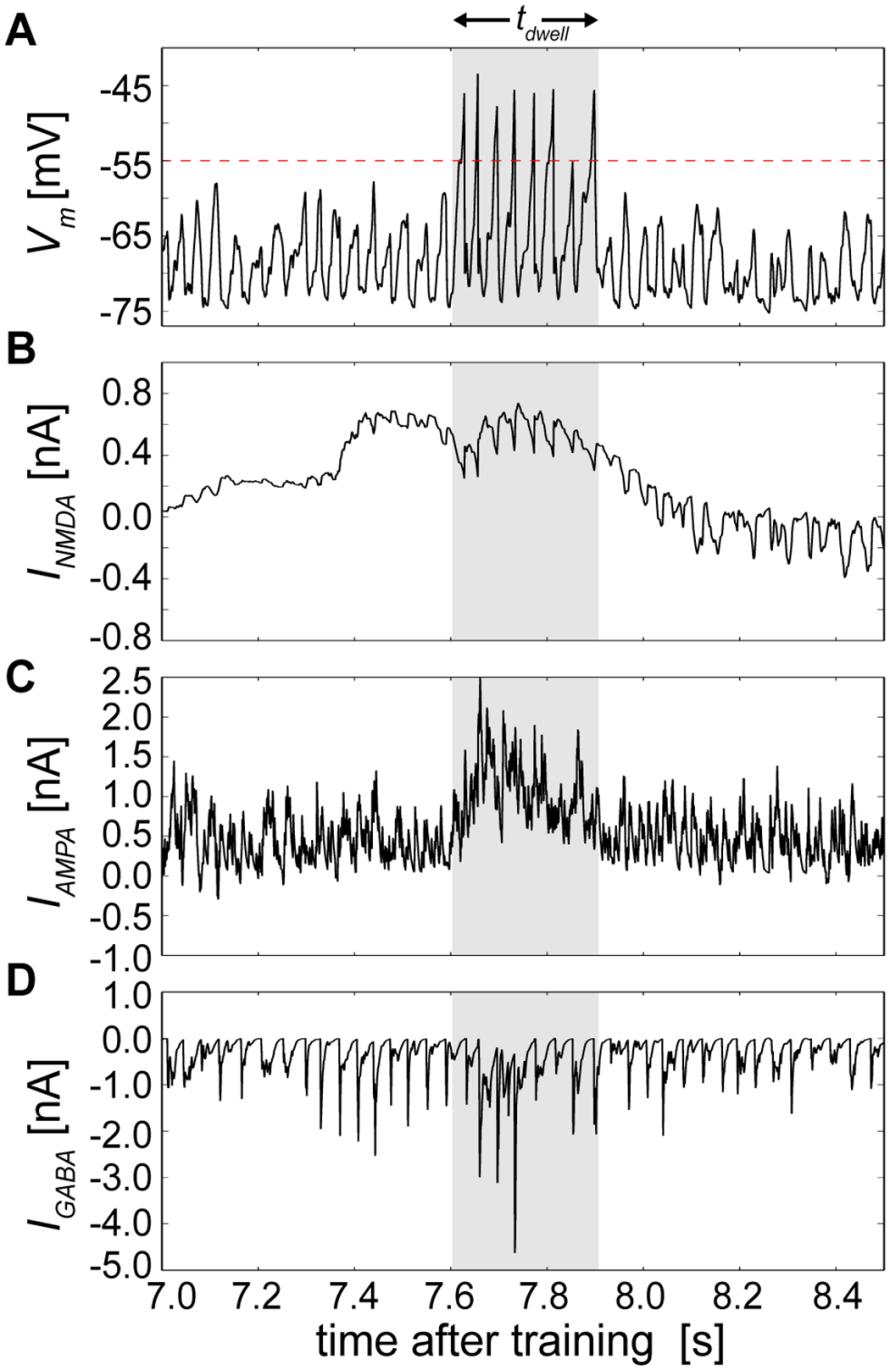
Upkeep of stable sequential state switching during recall is permitted by the interplay of excitation and inhibition in a single neuron. **(A)** Recorded *V*_*m*_ of a randomly chosen single cell whose period of increased tendency for being suprathreshold coincided with its brief engagement within an attractor. The red dotted line represents the membrane voltage threshold *V*_*t*_ for reference, and the shaded area represents a detected attractor state that is reproduced in B-D. **(B)** The same cell’s net NMDA current, which combined positive and negative afferent BCPNN weights. **(C)** The same cell’s net AMPA current, which combined positive and negative afferent BCPNN weights. **(D)** The same cell’s GABA current originating from local basket cell inhibition.

As the network with IPI = 0 ms proceeded through sequential states, individual neurons not actively participating in attractors were mostly quiescent, being suppressed through lateral inhibition. Previously referred to as the background state [22], single cells displayed characteristically hyperpolarized *V*_*m*_ (Fig 6A, see Eq 6 in Methods) until recruited into an attractor, where they then received an excitatory push through delayed activation of the NMDA receptor (Fig 6B, Eq 7). Companion neurons then followed, and there was a period of mutual excitation that stabilized attractor activity via the AMPA receptor (Fig 6C, Eq 7). Once reaching fruition, the attractor immediately began to perish due to ongoing cellular adaptation and short-term depression (Fig 3J and 3K, Eqs 9 and 11) that ensured its neurons would eventually lose their competition against neurons of opposing attractors due to GABA receptor activation (Fig 6D, Eq 7).

On the network level, the effect of systematically eliminating one dynamical mechanism at a time provided insights into each of their overall contributions to the circuit (S4 Fig). Eliminating plastic mechanisms one at a time each degraded sequence replay capabilities except for intrinsic plasticity. Most mechanisms also degraded sequence replay capabilities at higher parameter settings with the exception being adaptation, whose detrimental effects were mitigated by its relatively short time constant, which remained constant for these experiments (see Eq 6 in Methods).

Introducing a variable *P*(switch) that determined the probability that individual sequence elements within a training pattern order would differ from the original one led to a disruption of structure in the terminal $g_{ij}^{NMDA}$ profiles (S5A Fig). The chance that a sequence element was switched was applied indiscriminantly for all sequence elements in all epochs. This alteration gradually degraded the capability of the network to perform sequential recall, similar to the effect of increasing the IPI (S5B Fig, compare to Fig 5B).

On the spiking level, a bimodal distribution of pyramidal cell inter-spike intervals (ISIs) representing either ISIs within attractor states or ISIs between attractor states arose (S6A Fig). The variability of single cell spiking was predominantly high for both pyramidal and basket cell spike trains, as reflected by their average local coefficient of variation of ISIs that clustered close to unity (S6B Fig).

**Fig 7:**
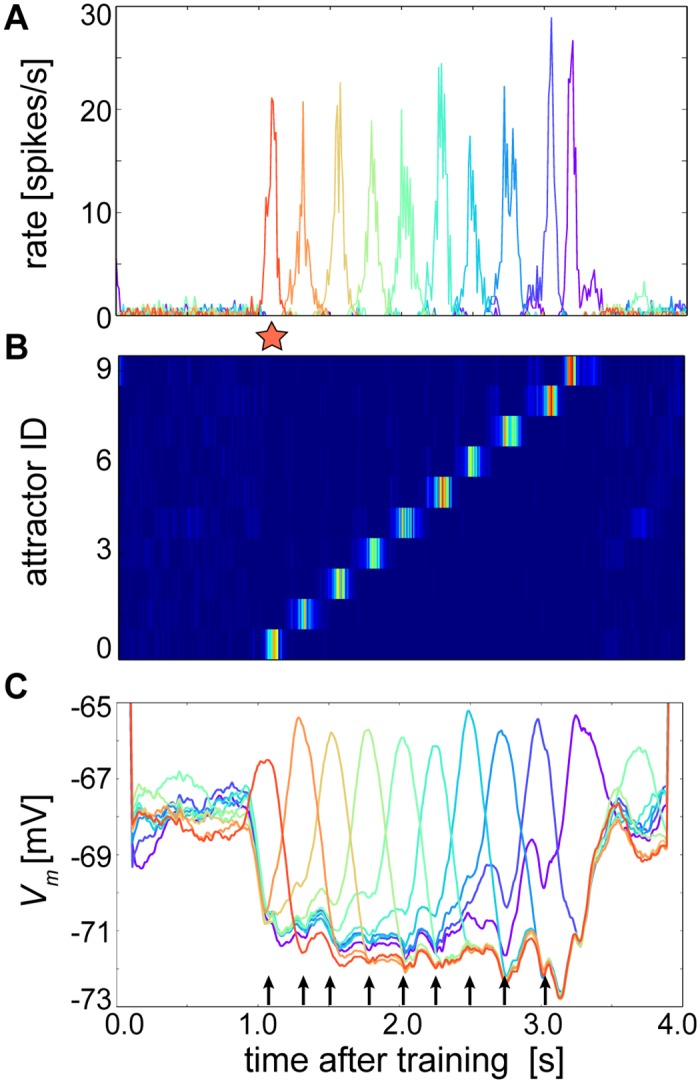
Rank ordered ramps of *V*_*m*_ depolariziations forecast the serial position of upcoming attractors during cue-triggered recall of a temporal sequence. **(A)** A cue (red star) presented 1 second into recall resonates through the trained network. **(B)** Activity levels based on (A) and sorted by attractor membership as in Figs 3H and 4D. **(C)** Average *V*_*m*_ (1 ms bins) taken for all excitatory cells in the network and smoothed per attractor index by a moving average with 200 ms window length. Truncated *V*_*m*_ values at the beginning and end are artifacts of the moving average procedure. In this example both before and after the cued sequence, patterns spontaneously but weakly activated, which could occur randomly due to the sensitivity of the system. Black arrows represent time periods occurring after the midpoint of the cue initially and after the midpoint of each attractor thereafter, during which the average *V*_*m*_ of the upcoming attractors are ranked according to their relative serial order within the sequence.

Up to this point, the network has been presented in a regime where background excitation was high enough to spontaneously rebroadcast stored patterns. To instead demonstrate cue-triggered recall of temporal sequences [43], the network input and training protocol were slightly altered by decreasing background excitation and adding temporal gaps between the last and first patterns of the training sequences. When briefly presented with a cue (see Methods) at the first pattern, the network could then perform sequential recall in the order that previously experienced conditioning stimuli were encountered (Fig 7A). Until presented with a cue and after recall, the network was generally disengaged and fired at low rates, exhibiting similar characteristics to the ground state that arose in Fig 3 (Fig 7B).

A notable aspect of Fig 6B was the elevated level of *I*_*NMDA*_ just before the cell entered an attractor state, suggesting that a neuron’s subthreshold dynamics could anticipate its future participation in the state despite the strong lateral inhibition that prevented it from spiking. As switching between states was not necessarily visible from the perspective of the single cell, *V*_*m*_ was averaged over all cells in the network for the cue-triggered simulation, from which it became clear that groups of cells exhibited consecutively postponed upward *V*_*m*_ transients corresponding to their anchoring events (Fig 7C).

Prior to cue presentation, averaged attractor membrane potentials spontaneously fluctuated yet roughly kept a reverse ordering of the original trained sequence. This was a relic from small leftover differences in $I_{\beta_{j}}$ after training because the last pattern was the one most recently presented and the first was the least recently presented to the network (Fig 3D; although referring to the non-sequentially trained network, the same principle holds). However once the cue was presented, and as similarly shown in Averbeck *et al*. (2002), the network dynamics were overwritten. The rank ordered attractors reorganized to correspond to the serial order of the trained stimuli, the subsequent attractor began to rise at roughly the middle of the current attractor, and the previous and subsequent attractors began to cross at approximately the point where the execution of the subsequent attractor began.

These relationships were most prominent for the first four attractors occurring directly after the cue (attractor index 0). The rank ordered *V*_*m*_ of attractors with indices 1–4 at this time corresponded to their trained serial order, but this ordering principle was not as well-defined at the same point in time for the more distant ahead attractors with indices 5–9. This was due to the limited temporal extent of $\tau_{z_{i}}^{NMDA}$ that was able to reach ahead only by a dwell time of about four attractors or so, after which its impact was unfelt. Relative rank ordering was not unique to the cue time, but was largely conserved even for attractors that occurred later on in the sequence. For example during the activation of attractor 5, the next highest ranks represented were attractors 6, then 7, then 8, and finally 9. Alterations of the moving average window duration did not qualitatively distort these properties.

But beginning around attractor index 5, the tendency of the subsequent attractor to rise at the middle of the current attractor, and the tendency of the previous and subsequent attractors to cross where the subsequent attractor began, broke down. For example, the attractors with indices 8 and 9 already began to rise while the attractor with index 6 was active. This was due to neural and synaptic fatigue whose effects become more pronounced in the network towards the end of the cue-triggered sequence, making it easier for neurons of attractors occurring later in the sequence to prevail in their competition with exhausted ones that were active earlier. Since the NMDA current is subject to temporal summation, later attractors also tended to rise earlier due to NMDA build-up from previously active inputs. Such considerations may inform future experiments evoking sequential memories whose temporal extent stretches beyond the time course of NMDA receptor decay.

### Factors Determining Temporal Sequence Recall Speed

In Fig 7A, the slow increase of average subthreshold *V*_*m*_ predicted the neuron’s participation within the approaching assembly, raising the question as to what cellular and network factors initiated this *V*_*m*_ build-up, determined the rising angle of its positive slope, and therefore increased or decreased the speed of ensuing attractor activation. At the network level, differential attractor dwell times would manifest as changes in the speed of sequential transitions.

**Fig 8:**
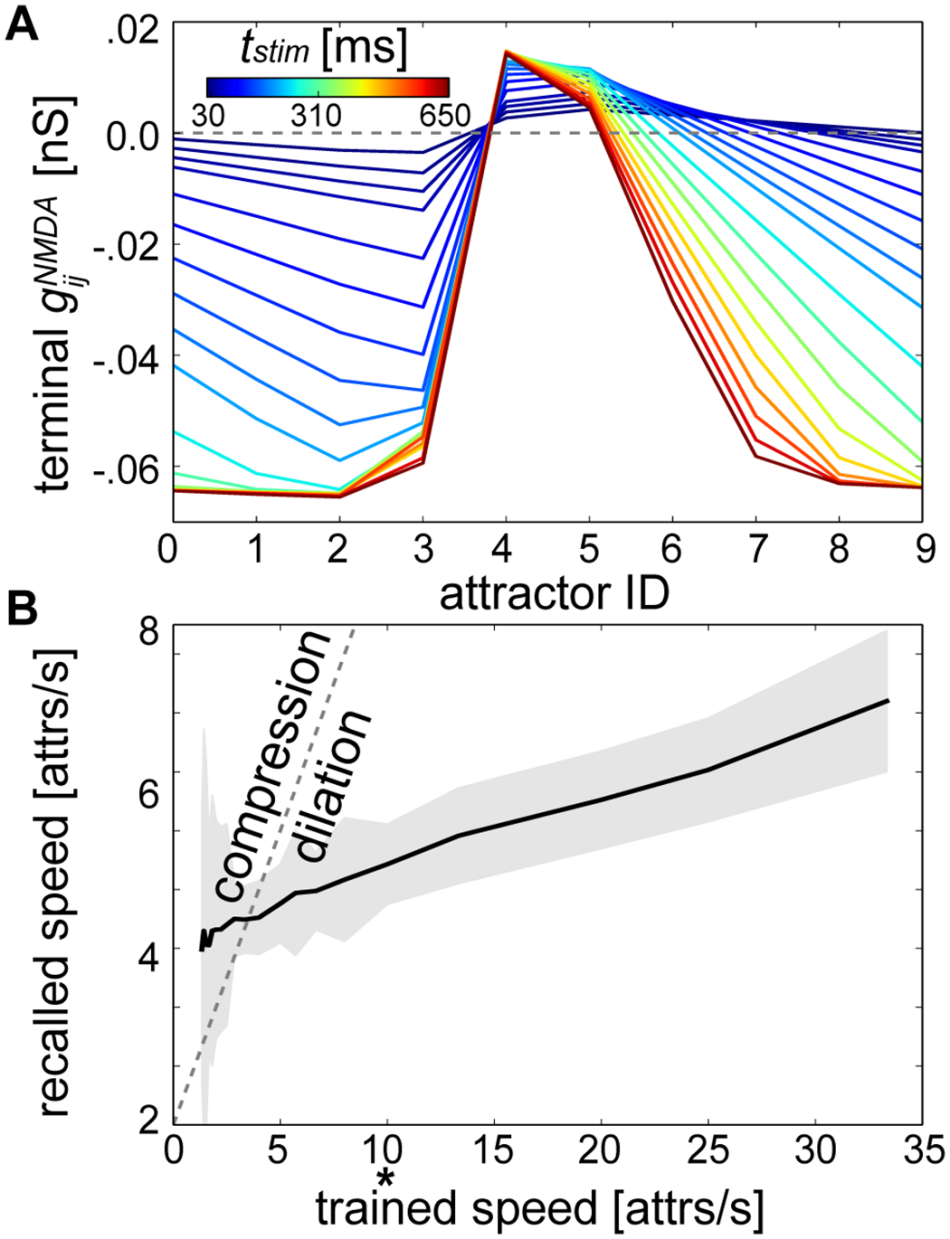
Dependence of temporal sequence speed on the duration of conditioning stimuli. **(A)** Average $g_{ij}^{NMDA}$ after training as in Fig 4C (reproduced here by the 100 ms line). **(B)** Speeds computed for the networks from (A) after 1 minute of recall. The dotted gray line represents a linear relationship between training and recall speeds (compression factor = 1), *t*_*stim*_ used previously can be discerned as * for reference, “attrs” abbreviates attractors and the shaded areas denotes the standard deviation.

To analyze which factors played a role in determining the relative lifetimes of sequential attractors, we began by taking the perspective that the speed at which sequences were learned was a product of the speed at which conditioning stimuli were presented to the network. Given its functional importance for sequence development in this model (see Fig 5), the strength of terminal NMDA weights are shown for different stimulus durations (Fig 8A). As *t*_*stim*_ was decreased, the shape of the terminal NMDA weight distribution flattened out, leading to an increased level of overall NMDA excitation and therefore faster network recall speeds (Fig 8B, see Eq 11 in Methods). We also measured the strength of terminal NMDA weights by varying the number of training epochs while *t*_*stim*_ = 100 ms (S7 Fig). Terminal weights could evolve to a relatively stable distribution of values after only 10 epochs, lowering the possibility that observed speed effects were localized only to the 50 epoch condition.

The speed with which attractors were recalled ranged from 3 to 7 attractors per second due to changes in *t*_*stim*_. But recall speeds were mostly dilated, displaying a compression factor < 1 (see Eq 12). This meant *t*_*stim*_ changes (i.e. trained speed changes) alone did not suffice to keep an even pace with the recall speed, let alone account for compression factors as high as those from experimental measurements [12,13,43].

**Fig 9:**
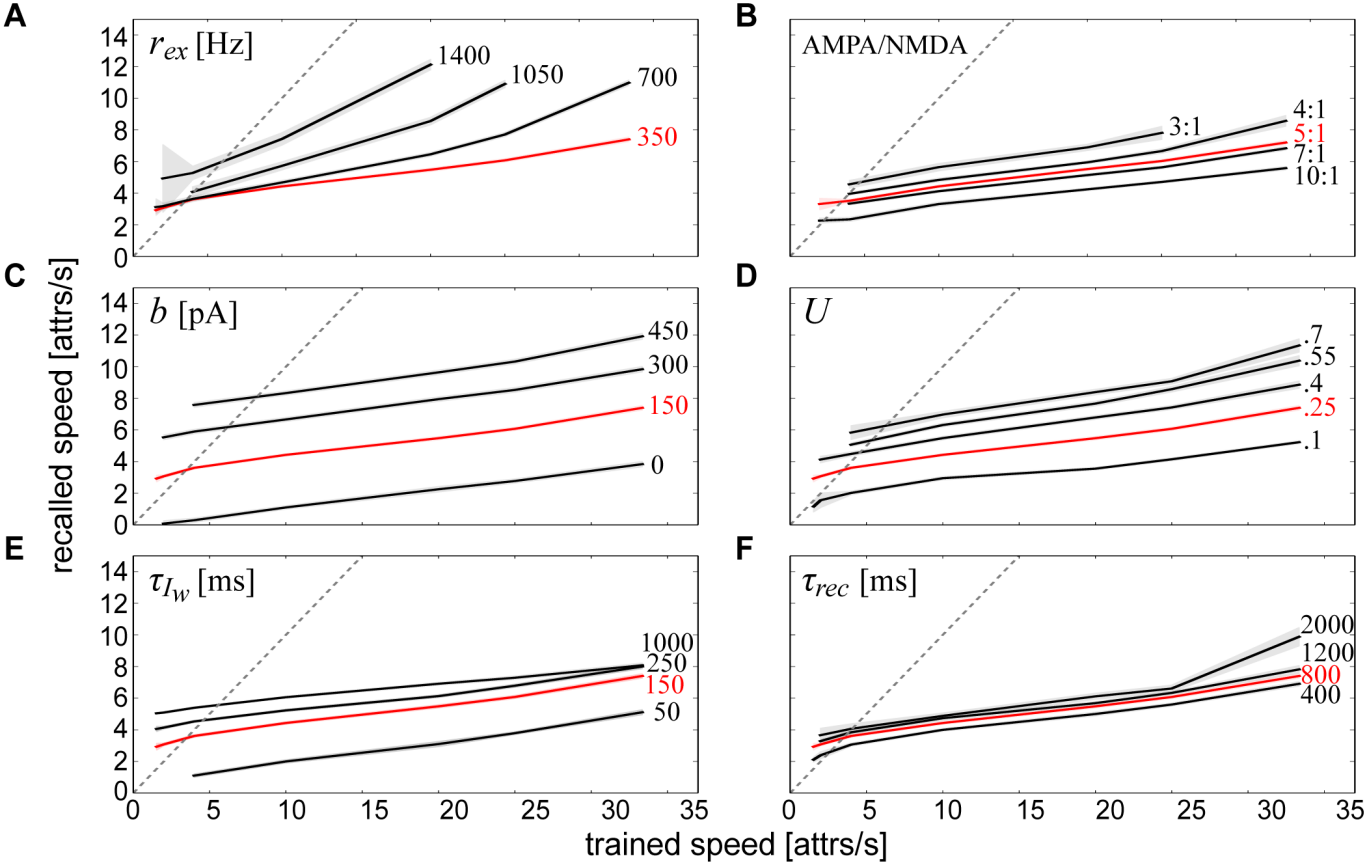
Multiparametric intrinsic determinants of temporal sequence speed. Univariate effects on recall speed are quantified by modulating **(A)** the rate of background excitation, **(B)** the AMPA/NMDA ratio, **(C)** the magnitude of neural adaptation, **(D)** the magnitude of short-term depression, **(E)** the time constant of neural adaptation and **(F)** the time constant of short-term depression. Simulations using the parameters from Fig 8B were reproduced in A-F in red for comparison. The dotted gray line represents a linear relationship between training and recall speeds (compression factor = 1, see Eq 12), “attrs” abbreviates attractors and shaded areas denote standard deviations.

An alternate hypothesis is that speed is not constrained as much by peripheral events as it is by intrinsic cortical mechanisms. When the network recalled sequences, it was apparent that the *V*_*m*_ of each attractor displayed weak subthreshold oscillations (see Figs 6A and 7C). This suggests that elevating *V*_*m*_ should lead to faster replay. To explore this possibility, we modified several different intrinsic parameters of the network after training and measured their effects on recall speed. The most straightforward modification we considered was to increase the amount of background noise in the network *r*_*ex*_, and as expected, delivering higher input levels to the excitatory cells accelerated recall speeds (Fig 9A).

Next, we considered the effect of synaptic currents on recall speed. We did not alter the absolute AMPA or NMDA synaptic efficacies alone, but rather altered their relative contributions via $w_{gain}^{syn}$ (Eq 4). Decreasing the AMPA/NMDA ratio led to a noticeable speed-up (Fig 9B), highlighting again the importance of NMDA in fueling the ascent of forthcoming attractor states.

Because the gradual erosion of attractor states was determined by a combination of neural adaptation and short-term synaptic depression (see Fig 3J and 3K), we next varied these dampening mechanisms in isolation to see how they affected sequence recall speed. We varied two types of parameters in the models of adaptation and short-term depression (see Eqs 9 and 11): their respective magnitudes, *b* and *U*, and time constants, $\tau_{I_{w}}$ and *τ*_*rec*_. Increasing the magnitude of both neural (Fig 9C) and synaptic (Fig 9D) fatigue enhanced recall speeds. Increasing the time constants of both neural (Fig 9E) and synaptic (Fig 9F) fatigue also enhanced recall speeds, but to a lesser extent. Overall, these results not only illustrated the relationship between intrinsic network parameters and recall speed, but also demonstrated a level of robustness of the model to these parameter changes since the sequential function of the network remained intact.

**Fig 10:**
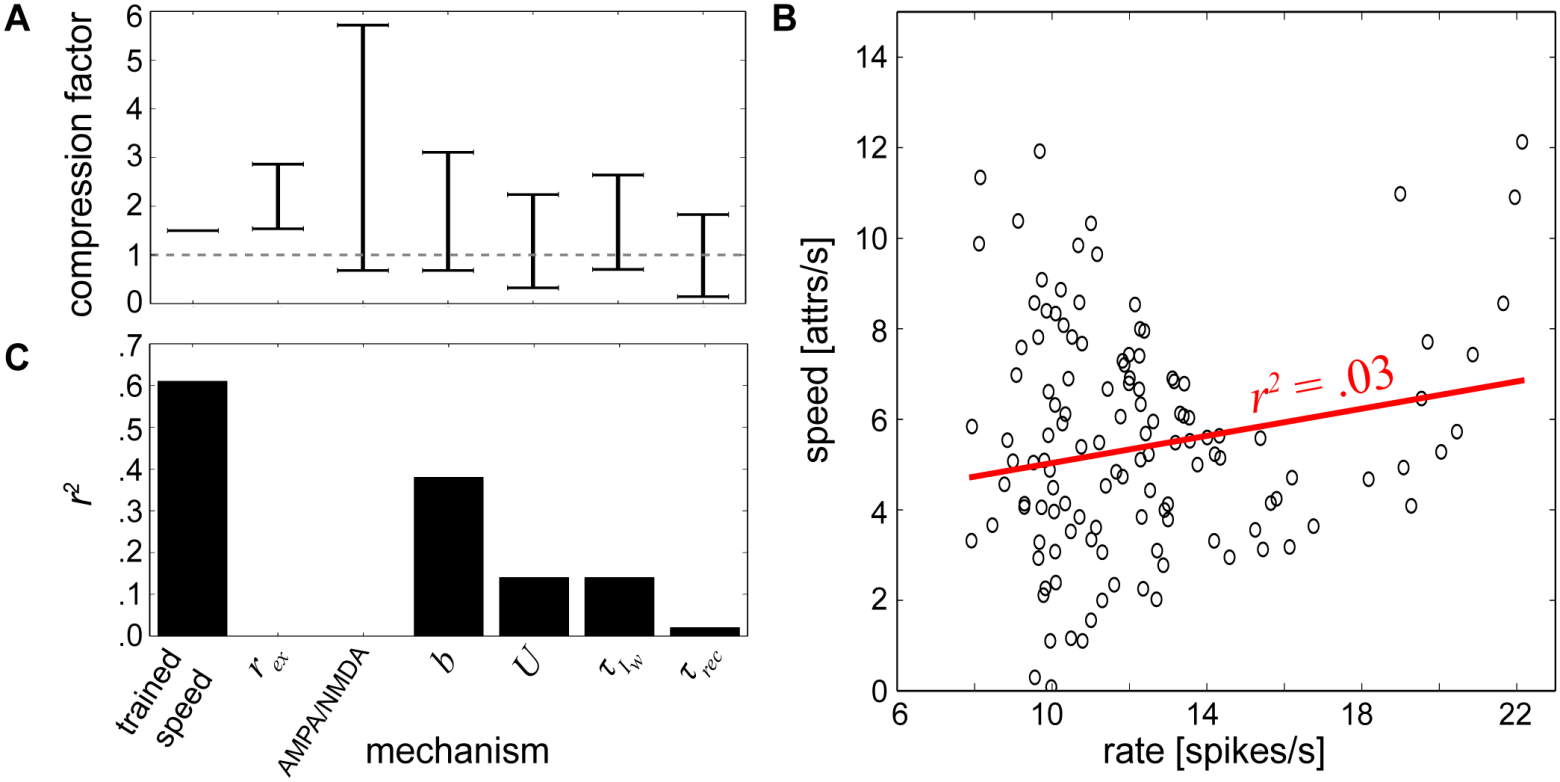
Characterization of speed changes. **(A)** Compression factor ranges measured for trained speeds of 2 patterns/second achieved by altering one parameter at a time and keeping all others constant. Horizontal bars denote compression factor cutoffs that were maximally allowable without violating edit distance tolerance levels, and the gray dotted line indicates a compression factor of 1. A second value does not exist for training speed since it was held at 2 patterns/second. **(B)** Cloud of points summarizing recall speeds and their corresponding firing rates from Fig 9. The linear regression line is displayed in red where *r*^*2*^ represents the square of the Pearson product-moment correlation coefficient of the two variables. **(C)** Correlation coefficients for individual mechanisms.

It became clear based on all of the parameter variations considered that temporal compression in this network was more easily attainable for longer stimulus durations (Figs 8B and 9). Since experiments demonstrating temporal compression similarly elicited firing activity at longer durations [12,13], we analyzed the extent to which the network could be maximally compressed and dilated for a pattern duration of *t*_*stim*_ = 500 ms (i.e. a trained speed of 2 patterns/second). Compression factors for each calibrated parameter at this trained speed are summarized by Fig 10A.

Although all of the parameters tested at this speed were able to alter the network speed to some extent, some more effectively than others, it would still be impossible to separate out which specific factor could give rise to compression if sequential activity was already compressed, for example in an experimental setting. To further characterize how these mechanisms could inform the network dynamics, we considered their effects on firing rates.

Linear regression analysis was performed on the relationship between firing rates and recall speeds using all of the output data procured from the different networks in Figs 8 and 9, and a weak relationship between these two variables was found (Fig 10B). Since there was minimal interaction between the mechanisms contributing to these variables, the same type of analysis was then performed for each individual network component (Fig 10C). We found that training speed and *b* were the most strongly correlated, *U* and $\tau_{I_{w}}$ were weakly correlated, and *r*_*ex*_, *τ*_*rec*_ and the AMPA/NMDA ratio were uncorrelated with average firing rate.

### Completion and Disambiguation of Overlapping Sequences

To further illustrate its robustness and functional flexibility, the network was trained using sequentially overlapping temporal stimulus patterns. Two separate training protocol were designed that were motivated by experimental paradigms [72,73] and adhered to generic sequential memory recall tasks [44]. For the following protocol, we reverted to a training configuration involving interposed delays between trials, in other words training blocks with IPI = 0 ms were separated by 2000 ms periods of quiescence as in Fig 7. Each protocol consisted of two unique subsequences of the complete sequence pattern alternatingly presented 50 times each.

**Fig 11:**
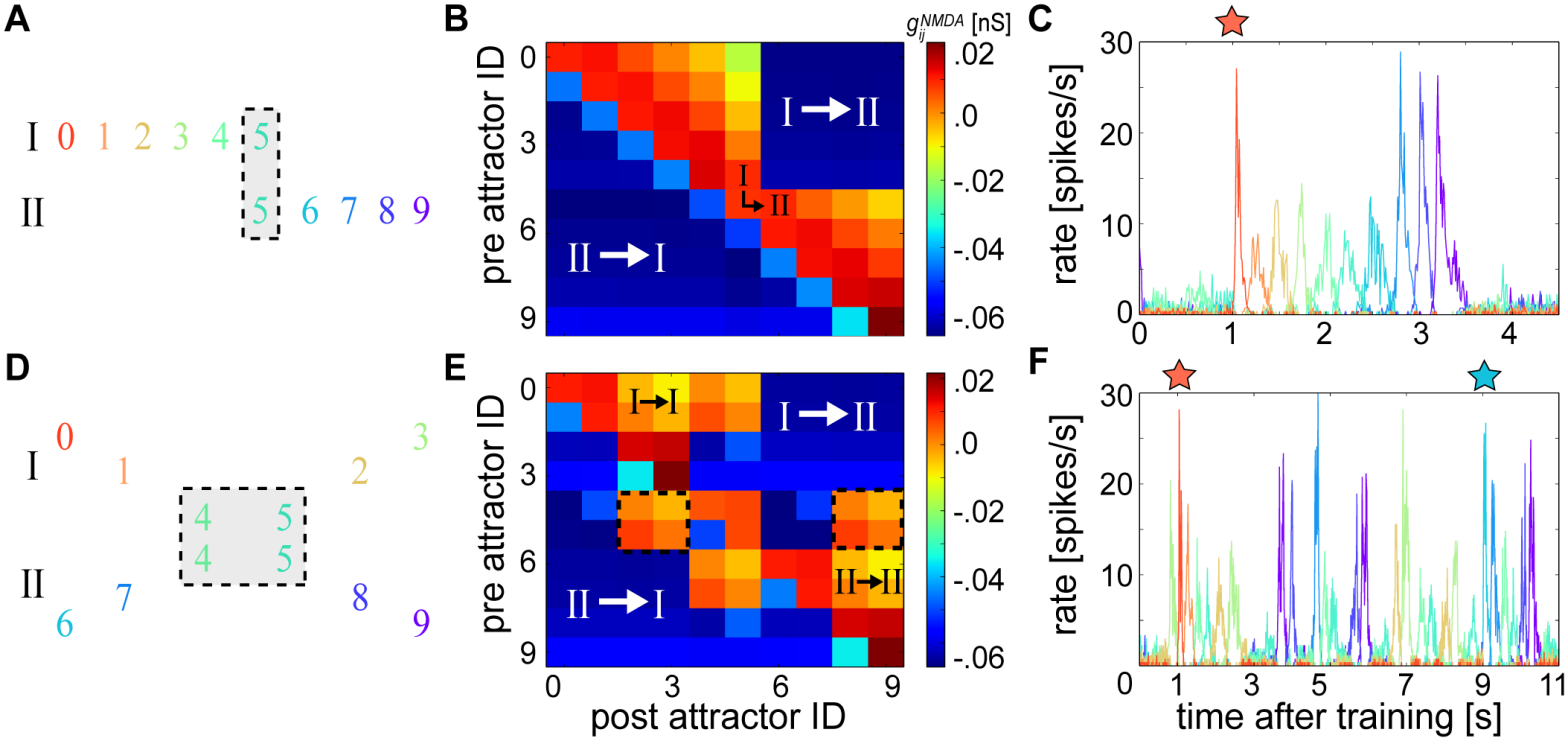
Cue-triggered overlapping sequence completion and disambiguation. **(A)** Schematic of sequential training patterns used to demonstrate completion. Roman numerals label uniquely trained subsequences that were alternatingly presented to the network, and the gray box highlights overlapping subsequence patterns. **(B)** Terminal average $g_{ij}^{NMDA}$ matrix resulting from (A). White Roman numerals identify regions of the weight matrix corresponding to learned connections between the non-overlapping subsequences of (A), which were reciprocally inhibiting. Black Roman numerals identify the crucial associations used for bridging the two subsequences together. **(C)** A cue (red star) presented to the first pattern 1 second into recall resonates through the network. **(D)** Schematic of the training pattern as in (A) demonstrating the problem of sequence disambiguation. **(E)** Terminal average $g_{ij}^{NMDA}$ matrix resulting from (D) and white Roman numerals as in (B). Here, black Roman numerals identify the crucial associations for bridging each individual subsequence together despite their shared patterns, which each form indistinguishable average connection strengths towards each branch of the fork as emphasized by the equivalent matrix cells contained within the dotted outlines. **(F)** Two separate cues (red and blue stars) presented 8 seconds apart each resonate through their corresponding subnetworks.

In the first test, alternating stimulus patterns consisted of two subsequences where the last pattern of the first subsequence matched the first pattern of the second subsequence (Fig 11A). The network should be able to bind the transitively associated discontiguous subsequences together and perform cue-triggered recall in its entirety despite the different degrees of temporal proximity between subsequences [73]. After training, the resulting NMDA weight matrix exhibited pronounced asymmetry (Fig 11B) as in Fig 4C. But nonspecific negative connections developed between all of the non-overlapping patterns such that the network could only rely upon the excitatory connections projecting to and from a single pattern in order to bridge the two subsequences together. Nevertheless, the network could overcome these limitations and successfully recall the entire sequence as in Fig 7 (Fig 11C).

In the second test, stimuli consisted of two alternating subsequences whose middle two patterns were shared between both subsequences (Fig 11D). The first two unique patterns of each subsequence were followed by an intersection where the next two patterns were shared by both subsequences; this was followed by a ‘fork’ that divided the subsequences again into two separate branches comprising their last two unique patterns [72].

The resulting NMDA weight matrix after training showed that the connections projecting from the overlapping patterns towards the patterns belonging to each fork were identical (Fig 11E). But the unique subsequence patterns occurring before the overlapping patterns selectively developed connectivity preferences towards the last two unique patterns of their associated fork branch, and formed negative projections towards patterns belonging to their opposing fork branch. These preferences, enabled by temporal extent of $\tau_{z_{i}}^{NMDA}$, allowed the network to successfully recall each individual subsequence when presented with its respective cue (Fig 11F).

Sequence disambiguation could be performed using a single overlapping pattern shared by both subsequences (S8 Fig), but not using more than two. Spontaneous attractors arose in between cue presentations during these experiments, but they did not hinder the ability of the network to faithfully perform recall. Due to the success of the task, the resulting CRP curve for this experiment was similar to the sequential one presented in Fig 5B, albeit with more noise outside of the lag 1 transitions due to these more unreliable spontaneous attractors (S9 Fig).

## Discussion

### Neurobiological Underpinnings

Spike-based Bayesian-Hebbian learning enabled imprinting of sequential memory patterns onto the neocortical microcircuit model. This convenient setup allowed us to scrutinize the cellular, synaptic, and network mechanisms underlying sequence formation. Although some of the generic mechanisms presented for learning temporal sequences here may be employed throughout other well-studied brain regions [74–76], the proposed model is identified with a neocortical microcircuit since the BCPNN derivation entails columnar processing [46,47].

Hypercolumns were locally governed by soft WTA dynamics, which is a generic neocortical organizational principle [49] that has been emphasized elsewhere in the context of sequential neural activity [77–79] and sparse distributed coding [45,80]. They were modularly organized into laterally inhibiting minicolumns (Fig 2) [37,81,82], which can be thought of as a more economical way for circuits to abstractly achieve a more dense and symmetric connectivity as originally postulated by the Hopfield model, given that connectivity is viewed at the minicolumn rather than cellular level [17]. Modularity is also present in the enthorinal cortex [83] suggesting some of the generic computational features of this model could be relevant in the context of grid cells, whose *V*_*m*_ modulations have been shown to closely match predictions from similar types of attractor models (Fig 7C) [84].

On the level of neurons and synapses, the learning rule incorporated several experimentally reported dynamical processes. We chose the AdEx model to demonstrate our findings because of its broad usage and availability in the simulation literature, its mapping between parameters and experiments [85], and its compatibility with recently developed neuromorphic hardware systems [86]. We would not expect employing other adapting neural models [87] to influence our results, since network dynamics are relatively robust to parameter changes and individual spikes. In fact, a reduced version of this model was recently run with generalized adaptive leaky integrate-and-fire neurons on the digital neuromorphic SpiNNaker architecture [88].

The intrinsic neuronal bias $I_{\beta_{j}}$ reflecting the prior activation level, was viewed as a model for the activity-dependent hyperpolarizing activation of A-type K^+^ ion channels mediated by Ca^2+^ regulated cationic currents [56] or TRP channels [57]. It shifted the input-output curve of the postsynaptic neuron such that persistently low active neurons received more hyperpolarizing current and highly active neurons received less hyperpolarizing current, in functional agreement with long-term potentiation of intrinsic excitability (LTP-IE) [55].

The proposed network does not require intrinsic plasticity in order to replay temporal sequences (see S4A Fig), but it is included here to comply with a previously introduced spike-based probabilistic framework [35]. The role of $I_{\beta_{j}}$ within the circuit depends on the distribution of training patterns. When the training patterns are presented equally often and at evenly spaced intervals, $I_{\beta_{j}}$ converges to similar values for all of the neurons in the network (Figs 3 and 4). But more complex training with patterns occurring at different frequencies (e.g. pattern 5 of Fig 11A and 11D) means that $I_{\beta_{j}}$ would be different for neurons belonging to different attractors. This opens up the possibility that rather than providing a generic downward pressure on activity, learned $I_{\beta_{j}}$ could more directly determine the sequence trajectory itself in some circumstances, although this was not observed in the simulations presented here.

Plastic changes in synaptic weights were determined by the relative window width set by $\tau_{z_{i}}^{syn}$ and $\tau_{z_{j}}^{syn}$ (see Fig 2B and S2 Fig). We set the postsynaptic *Z*_*j*_ trace time constants to $\tau_{z_{j}}^{AMPA}$ = $\tau_{z_{j}}^{NMDA}$ = 5 ms, reflecting the fast closing dynamics of AMPA channels and the membrane depolarization due to spike back-propagation, respectively [50]. The latter helps lift the Mg^2+^-block on NMDA channels and allows Ca^2+^ to flow into the postsynaptic neuron and induce synaptic potentiation upon activation of the NMDA receptor by glutamate [89]. Presynaptic *Z*_*i*_ trace time constants were set to $\tau_{z_{i}}^{AMPA}$ = 5 ms and $\tau_{z_{i}}^{NMDA}$ = 150 ms, the latter reflecting the slow closing dynamics of NMDA receptor gated channels.

AMPA synapses had symmetrical learning time constants $\tau_{z_{i}}^{AMPA}$ = $\tau_{z_{j}}^{AMPA}$ = 5 ms, which were matched to the AMPA synaptic time constant *τ*_*AMPA*_ = 5 ms. Learned auto-associative AMPA connectivity was responsible for sharpening and stabilizing activity within attractor states. But NMDA synapses had asymmetrical learning time constants, which enabled functionally asymmetric inter-pattern hetero-association. Asymmetric excitation (Fig 4C) can be achieved through synaptic learning [71,90], which may explain the development of asymmetric hippocampal place [91] and visual receptive [92] fields.

We matched the shape of the learning kernel to the shape of the EPSP (Fig 2) following previous studies [66–68], and also acknowledge this parameterization is sufficient but not necessary for temporal sequence recall in our model. In principle, it would also be possible using different degrees of NMDA window shape asymmetry, and even using a symmetrical NMDA kernel (e.g. by setting $\tau_{z_{i}}^{NMDA}$ = $\tau_{z_{j}}^{NMDA}$ = 150 ms). In a cue-triggered setup with trained symmetrical NMDA, the sequence could be recalled either forwards or backwards by presenting a cue to the first or last attractor, respectively. Instead of being directed by NMDA asymmetries, the preferred sequence trajectory would evolve according to adaptation and short-term depression. After shutting down an active attractor, the network would activate the immediately neighboring attractor since it would receive the next highest amount of learned potentiation.

However, if this were the case experimentally, one would expect cortical networks to be able to be trained on a specific stimulus order and then nonselectively perform sequential pattern completion regardless of whether the cue was presented at the head or the tail of the original sequence. Since it is rather the case that there is a high specificity for trained sequence order [43,93], we emphasize the functional importance of an asymmetrical NMDA kernel in this model. It should be noted that such temporal specificity is also compatible with what would be expected from STDP [92].

As apparent from Figs 3D–3F, 4B and S1 Fig, $g_{ij}^{syn}$ and $I_{\beta_{j}}$ did not continually grow nor shrink but ultimately settled into quasi-stationary values that reflected accrued knowledge of the stimuli. Such behavior is functionally reminiscent of synaptic scaling [94] and anti-homeostatic plasticity of intrinsic excitability [55], which can similarly stabilize Hebbian synaptic plasticity and LTP-IE over longer time scales, respectively.

Furthermore, as the *P*_*j*_ trace was involved in the computation of both $I_{\beta_{j}}$ and $g_{ij}^{syn}$, the learning model can be viewed in terms of co-expressed synaptic and nonsynaptic plasticity arising from shared biochemical signaling cascades [95]. The computational role of an eligibility trace (i.e. *E* trace) as an intermediate exponential filter in between the *Z* and *P* traces was previously explored [35], but was excluded here because delayed reward learning was not required for learning temporal sequences.

The globally applied ‘print-now’ factor *κ* controlled both synaptic and intrinsic plasticity. In contrast to learning scenarios in which the dynamics of the network disturb the training regime, our protocol activated *κ* and de-activated both *β*_*gain*_ and *$w_{gain}^{syn}$* during pattern presentation and vice-versa during recall (see Figs 3 and 4). Clamping is unconventional in spiking models, but it is more common in classical recurrent associative networks in which recalling old patterns actively interferes with learning new patterns. This dilemma has been addressed in spiking models by introducing multiple time scales with different synaptic states [96,97], but we assume here that neurophysiological mechanisms clamp activity to the patterns to be learned through selective suppression [61].

We consider this third-factor *κ* as representing the influence of a non-local neuromodulatory signal like acetylcholine or dopamine, which are thought to play important roles in the emergence of temporal sequential activity [93,98]. They can have pronounced postsynaptic effects on cortical neurons like increasing spiking activity from synaptic stimulation. These effects enhance the response of cortical neurons to afferent input during the suppression of synaptic transmission within intrinsic connections, enabling neurons to clamp to afferent input patterns [61].

Though beyond the scope of this work, activating *β*_*gain*_ and *$w_{gain}^{syn}$* during pattern presentation should not drastically diminish the network’s learning capabilities under most circumstances. A suppression of competing minicolumn activity would largely prevent neurons outside of a presented pattern to be recruited into that pattern, as long as patterns remained relatively orthogonal. Introducing more overlapping patterns would likely re-establish the need to modulate *κ*, *β*_*gain*_ and $w_{gain}^{syn}$. Furthermore, we applied a simplified binary model of *κ*, but acknowledge that introducing more realistic neuromodulatory dynamics [99] could also be a productive direction for future study.

Both positive and negative weights can be learned in the BCPNN formulation due to its Bayesian derivation. Negative weights are neurobiologically interpreted as a form of disynaptic inhibition (see Fig 1B) [62–65]. In the interest of more biologically detailed setups, networks with negative synaptic weights have been shown to be functionally equivalent to ones with both excitatory and inhibitory neurons with only positive weights [100]. Introducing explicit fast-spiking linear interneurons may influence the network dynamics due to their added synaptic delays, but these effects would be minimal since our model is not critically dependent upon precise spike timing.

### Temporal Sequence Dynamics

Supporting the notion that learned spatiotemporal sequences depend on stimulus timing (see Fig 8), evoked responses are sensitive to the duration of experienced temporal patterns *in vitro* [40], are spontaneously replayed at the same speed they are trained on *in vivo* [101], and are decreased for identically ordered but re-timed stimuli down to a difference of just 50 ms [93]. But there is a growing consensus that the involvement of peripheral or environmental signals cannot fully account for the speed of sequential activity [102]. Supporting the notion that internally generated signals play an important role (Fig 9), sequence speeds may be altered depending on the behavioral state of the animal [43] and in the absence of any perceivable input [5]. Furthermore, they are not time-locked to the onset of the stimulus [4] or task [9], and they are compressed during sleep in visual [13,43] and prefrontal [12] cortices.

Although some studies of cortical activity report variable sequence element durations [4,8], they have previously been shown to last about 100 ms and correlate with the animal’s reaction time [3]. They can also last up to hundreds of milliseconds and dynamically vary as a function of the behavioral relevance of spatial information [5] or moment within task execution [1]. Temporal compression is particularly important for memory networks since patterns with shorter durations are more amenable to potentiation through associative processes (Fig 8A). Indeed, learned stimulus sequences replay up to an order of magnitude faster than trained time in prefrontal cortex [12] and primary visual cortex [41,43].

Conceptually, our model provides a way to explore how spike-based processes could influence sequence replay speeds (Fig 9). Several aspects including the amount of short-term depression [103], differential contributions of AMPA and NMDA currents [104], and strength of adaptation currents [105], are all considered to be under the influence of different neuromodulators. Their demonstrated effects on replay speed in our model imply that sequential circuit activity could be dynamically modified through neuromodulation [93,98]. The wide diversity of published values associated with these mechanisms [58,106], along with the functional heterogeneity inherent within neocortical microcircuits [3,107], lent some leeway to our choice of biophysical parameter ranges while measuring replay speeds. We expect that the addition of physiological detail to the model in the form of more diverse interneuron subtypes would influence speeds as well [108,109].

The generic neural mechanisms elucidated by this model may also be suitable for describing temporal interactions in the hippocampus, where its well known that place cell activity can be compressed [41] or dilated [110] in time. A functionally powerful aspect of our model is that it can recall sequences in reverse by switching $\tau_{z_{i}}^{NMDA}$ and $\tau_{z_{j}}^{NMDA}$ (Fig 4G). Although bidirectional replay is typically associated with hippocampal place cell activity [41,42], alterations in plasticity window shape between groups of reciprocally connected neurons may also constitute a more generic learning mechanism for evaluating past experiences.

While orthogonality can reduce interference between patterns and lead to pristine synaptic weight matrices (Figs 3 and 4), natural stimuli often consist of common repeating patterns, which would in turn shape how sequences are memorized by the network [111]. That neural networks must solve the problems of overlapping sequence completion and disambiguation was proposed on theoretical grounds [44] and confirmed later on by experiments in neocortical [72,73] and hippocampal [112,113] preparations. The essence of the problem boils down to ambiguous information: is the circuit able to distinguish separate events that share common repeating patterns and remember earlier patterns in order to complete sequence patterns despite overlapping interference?

We have demonstrated examples of overlapping sequence recall inspired by these experiments (Fig 11) that may also be relevant for working memory tasks in which divergent sequential trajectories unfold when the animal’s travel routes share common spatial locations during training (e.g. the center arm of a T-maze) [9,11]. In this way, our simulations support a functional architecture in which overlapping neural assemblies form coherent network states that can be selectively deployed depending on the cognitive requirements of the task at hand [10]. Indeed due to the extended time course of NMDA receptor dynamics, the model can disambiguate up to 2 overlapping patterns (Fig 11D–11F, S9 Fig). Given the many other possible combinations of overlapping and nonoverlapping sequence configurations that could be simulated using this network model, future work could address scaling the network up in order to accommodate longer and even more complex hetero-associative patterns.

### Related Theoretical Models

Presumably in an effort to avert analytical intractability, models of sequence generation typically rely only upon one or few underlying computational mechanisms. But large-scale, recurrently connected cortical circuits exhibit complex dynamical interactions; they play host to many plastic mechanisms that intricately sculpt and are sculpted by ongoing activity. A key question that arises then is how to balance the presence of such generic and widespread phenomena while reducing unnecessary model complexity.

Early models enabled Hebbian learning of attractor sequences using a fully connected network of binary neurons [114] and a slow synaptic refresh mechanism in more realistic rate-based neurons [115]. In conjunction with STDP, more recent approaches have proposed a global astrocytic factor [67], homeostatic intrinsic plasticity [34], homeostatic synaptic plasticity [116], presynaptic homeostatic synaptic plasticity [117], homeostatic synaptic and non-Hebbian heterosynaptic plasticity [118], homeostatic synaptic and intrinsic plasticity [78], and homeostatic synaptic and homeostatic intrinsic plasticity [119]. In the same vein, our model combines synaptic and nonsynaptic plasticity mechanisms to hypothesize a means of producing sequences.

The excitatory-to-inhibitory and inhibitory-to-excitatory WTA connections (see bottom of Fig 1A) are predefined in this model. WTA dynamics can theoretically be stably learned in rate-based networks of randomly connected neurons using both excitatory and inhibitory plasticity [120]. We speculate that introducing Hebbian excitatory-to-inhibitory and inhibitory-to-excitatory WTA synapses and subsequently nonspecifically stimulating all inhibitory cells during the presentation of each element of the sequence may permit similar dynamics. In line with several newly proposed spiking models that enable plasticity for all synaptic connections by relying upon inhibitory plasticity to learn and retrieve memories [118,121,122], learning WTA synapses in our modular model is a promising direction of future study.

Our model avoids unnecessary complexity by framing the accompanying dynamical changes in the context of Bayes’ rule [35]. Spike-based BCPNN computes synaptic weights and intrinsic biases based upon probabilities that are estimated by cascades of interacting synaptic and nonsynaptic memory traces. Supporting the view that cortical networks can represent probability distributions that are interrelated via Bayes’ rule [123–126], BCPNN statistically interprets glutamate receptor kinetics as establishing a window of temporal integration for Bayesian-Hebbian learning.

Bayesian inference is a viable neurobiological encoding strategy that reasonably captures numerous psychophysical [127] and even electrophysiological [124,128] data. Furthermore, it is attractive from a computational point of view due to its ability to represent and compute using uncertainty in a principled way [123]. Uncertainty is inherent in situations where there are several plausible interpretations of an incoming signal, for example on a macroscopic level given noisy or incomplete sensory stimuli, or on a microscopic level where stochastic biochemical processes abound [129,130].

By the same token, the intuition that probabilistic computation should occur on the timescale of a single ISI in order to match the temporal scale of perception and sensorimotor integration [125] has propelled theoreticians to devise biophysical substrates for the neural implementation of Bayes’ rule. Like the aforementioned sequence studies, such models tend to consolidate multiple generic learning processes into a single cohesive probabilistic framework [35,68,131,132].

Likewise, and by virtue of the postsynaptic traces (i.e. *Z*_*j*_ and *P*_*j*_) and third factor *κ*, our approach highlights how synaptic, intrinsic and neuromodulated plasticity mechanisms [133,134] spanning several time scales [96,135] could interact to memorize sequential patterns of activity. Moreover, the incorporation of cellular adaptation [85] and synaptic depression [103] for terminating attractor states comprehensively provides an explicit function for these other dynamic mechanisms in the proposed network’s overarching computation. It conforms to previous work asserting an essential role for NMDA [136], adaptation [137] and short-term plasticity [9,138–140] in shaping sequential network dynamics over time.

Generation of sequential states in temporally asymmetric attractor networks was proposed early on [38,39]. The innovation in our work involves learning temporally asymmetrical NMDA conductances and temporally symmetrical AMPA conductances between spiking neurons. Unique propagatory roles for AMPA and NMDA receptors in terms of circuit activity are suspected [141], and it is not surprising that the long decay time constant of NMDA would contribute to sequential neuronal activity [40,43,98] given its neural ubiquity, along with its postulated role in long-term memory [142], working memory [143], predictive coding [144,145], and storing hetero-associative sequence information [146]. Its prolonged activation acts as a slow memory trace that dynamically bridges different neuronal assemblies [18,147], allowing passage of representational content from one group of neurons to the next in sequence, and propelling the network along a trajectory through state space.

Sequential activity due to asymmetrical connections has alternatively been described in the context of reservoir computing [148], where the trajectory through state space via transient dynamics emphasizes a departure from classical attractor networks involving the inevitable convergence to a single state [149]. However, active states in the model presented here should be distinguished from fixed-point attractors since they exhibit finite dwell times before being repulsed by adaptation and short-term depression. In our model, the circuit undergoes dynamic meta-stable patterns of activity whose voyage through state space is dictated by the extent of previously learned temporal associations, offering a plausible way in which time may be implicitly encoded within an attractor network.

## Methods

### Neuronal and Synaptic Models

Hypercolumns were spatially laid out on a 3x3 square grid with coordinates (*m*, *n*) {0, 1, 2}. The axonal delay *t*_*ij*_ for each BCPNN connection between presynaptic neuron *i* and postsynaptic neuron *j* was set according to hypercolumn membership of the neurons using the Euclidean distance between corresponding hypercolumn grid coordinates:

$$\bar{t_{ij}}=\frac{d_{norm}\sqrt{{\left(m_{i}^{}-m_{j}^{}\right)}^{2}+{\left(n_{i}^{}-n_{j}^{}\right)}^{2}}}{V}+1\text{ ms, }t_{ij}\sim N(\bar{t_{ij}},\;.1\bar{t_{ij}}).$$ (5)

Delay values were randomly generated from a normal distribution with standard deviation set 10% relative to their mean $\bar{t_{ij}}$ in order to account for individual arborization differences. The conduction velocity *V* was 0.2 mm/ms, and we assumed the 3x3 piece of cortex occupied a 1.5x1.5 mm area so setting *d*_*norm*_ = 0.75 mm scaled relative distances accordingly. The additional offset of 1 ms was included so that the shortest delays in the network, which occurred when neurons *i* and *j* belonged to the same hypercolumn (i.e. *m*_*i*_ = *m*_*j*_ and *n*_*i*_ = *n*_*j*_), were centered on 1 ms.

All simulations were conducted using the NEST simulator [150] with the AdEx neuron model [85]. The AdEx model was modified here in order to account for the intrinsic excitability term *β*_*j*_, and we simplified the model further by eliminating subthreshold adaptation dynamics. For each neuron, the temporal evolution of the membrane potential *V*_*m*_ and adaptation current *I*_*w*_ obeyed:

$$C_{m}\frac{dV_{m}}{dt}=-g_{L}(V_{m}-E_{L})+g_{L}\Delta_{T}e^{\frac{V_{m}-V_{t}}{\Delta_{T}}}-I_{w}(t)-I_{tot}(t)+I_{\beta_{j}}+I_{ext},\;\tau_{I_{w}}\frac{dI_{w}}{dt}=-I_{w}.$$ (6)

The model theoretically describes spike emission at the time when *V*_*m*_ diverges towards infinity, but in practice, when a threshold *V*_*t*_ = -55 mV was approached (*V*_*m*_ ≥ *V*_*t*_), *V*_*m*_ was reset to *V*_*r*_ = -70 mV. Here, *V*_*t*_ is not a strict threshold per se, but rather the center of an exponential nonlinearity modeling each action potential upswing. At the same time as this discrete voltage reset, the adaptation current *I*_*w*_ increased according to *I*_*w*_ = *I*_*w*_ + *b* with *b* = 150 pA. This was followed by a decay with time constant $\tau_{I_{w}}$ = 150 ms. Unlike pyramidal cells, local basket cells included neither spike-triggered adaptation (*b* = 0) nor intrinsic excitability (*β*_*gain*_ = 0). *V*_*m*_ was initialized to random values between *V*_*t*_ and *V*_*r*_ for each neuron. Otherwise, all neurons had identical parameters.

The total current flow across the membrane was determined by the membrane capacitance *C*_*m*_ = 280 pF, leak reversal potential *E*_*L*_ = -70 mV, leak conductance *g*_*L*_ = 14 pS, spike upstroke slope factor Δ_*T*_ = 3 mV, $I_{\beta_{j}}$ defined by Eq 4, external input *I*_*ext*_ set by Poisson noise (see next subsection), and total synaptic input current to postsynaptic neuron *j* originating from other presynaptic neurons in the network:

$$I_{tot_{j}}(t)=\sum_{syn}\sum_{i}g_{ij}^{syn}(t)(V_{m_{j}}-E_{ij}^{syn})=I_{AMPA_{j}}(t)+I_{NMDA_{j}}(t)+I_{GABA_{j}}(t).$$ (7)

The definitions of *I*_*totj*_(*t*), *V*_*mj*_ and $E_{ij}^{syn}$ carry over from Eq 6 but now assume the perspective of pre- and postsynaptic neurons *i* and *j*, and synaptic conductance changes $g_{ij}^{syn}(t)$ defined below (see Eq 9) generate individual postsynaptic currents *I*_*AMPAj*_, *I*_*NMDAj*_ and *I*_*GABAj*_. Synaptic reversal potentials were set according to $E_{ij}^{AMPA}$ = $E_{ij}^{NMDA}$ = 0 mV and $E_{ij}^{GABA}$ = -75 mV. When the sign of the BCPNN weight turned negative, the inhibitory reversal potential $E_{ij}^{GABA}$ was used instead of $E_{ij}^{AMPA}$ or $E_{ij}^{NMDA}$.

In addition to the long-term BCPNN plasticity mechanisms, AMPA and NMDA synapses between pyramidal cells (i.e. $w_{ij}^{syn}$>0, connections with positive learning outcomes) were subject to short-term depression in a manner prescribed by the Tsodyks-Markram formalism [103]. In this model, a finite fraction of usable synaptic resources out of the total available is spent due to vesicle depletion from each incoming presynaptic spike at time $t_{sp}^{i}$:

$$\frac{dx_{ij}^{dep}}{dt}=\frac{1-x_{ij}^{dep}}{\tau_{rec}}-Ux_{ij}^{dep}\sum_{sp}\delta (t-t_{sp}^{i}-t_{ij}).$$ (8)

The parameter *U* = 0.25 controlled the portion of resources utilized in response to a spike, 0 ≤ $x_{ij}^{dep}$ ≤ 1 was the fraction of resources available and was exponentially replenished towards its baseline level (i.e. 1) between spikes with a time constant *τ*_*rec*_ = 800 ms, and *t*_*ij*_ was the distance dependent transmission delay (Eq 5).

A presynaptic input spike at time *t*^*i*^ originating from either recurrent or external sources evoked a conductance increase of $x_{ij}^{dep}(t)w_{ij}^{syn}$ nS followed by an exponential decay:

$$g_{ij}^{syn}(t)=x_{ij}^{dep}(t)w_{ij}^{syn}e^{-\frac{t-t^{i}-t_{ij}}{\tau_{syn}}}H(t-t^{i}-t_{ij}).$$ (9)

Here, *H*(·) represented the Heaviside step function. Weight values for the local static excitatory and inhibitory feedback connections within a single hypercolumn were drawn from a normal distribution with a standard deviation of 10% relative to their mean strengths, which were set to $w_{ij}^{AMPA}$ *~ N*(6.65, .665) nS and $w_{ij}^{GABA}$ *~ N*(33.3, 3.33) nS for AMPA and GABA synapses, respectively. The synaptic time constant for GABA synapses was set to *τ*_*GABA*_ = 5 ms. Parameters used in all network simulations can be found in S1 Appendix.

### Stimulation Paradigm for Attractor Learning

Spiking attractor memory networks can store static memory patterns and have been linked to cortical memory architecture and dynamics, including performance of perceptual operations like pattern retrieval, rivalry and completion [17]. Two types of attractor states coexist in such networks: a ground state displaying unspecific and non-selective low-rate firing, and several possible active states where one population displays an elevated foreground activity and the rest display a low-rate background activity. Lateral inhibition (Fig 1A) is primarily responsible for silencing neurons outside the active attractor, and attractor activity is terminated through neural and synaptic fatigue (Eqs 9 and 11) [45]. External stimulation or high amounts of background activity can transiently switch the network into one of the coding attractors. We use the term attractor to refer to the state displaying elevated coding foreground activity throughout this work.

The network was trained by selectively stimulating neurons belonging to each pattern in succession for a duration *t*_*stim*_ = 100 ms (but note *t*_*stim*_ is varied in Fig 8) through external presynaptic background sources (Eq 7) such that neurons in active patterns fired at *f*_*max*_ = 20 Hz. An epoch was defined as the period during which each pattern was stimulated once, and training consisted of 50 epochs. Simulating upstream network input, a constant amount of Poisson noise *r*_*ex*_ = 350 Hz through $w_{ij}^{AMPA}$ = 5 nS strength synaptic input was applied to each pyramidal cell during recall. These parameters determined the amount of external input *I*_*ext*_ (Eq 6). During cue-triggered recall experiments (see Figs 7 and 10), *r*_*ex*_ was reduced to 150 Hz, and instead, a stimulus consisting of a Poisson spike train with an average firing rate of 200 Hz through $w_{ij}^{AMPA}$ = 5 nS strength input synapses was delivered to each neuron of the given pattern for 100 ms. The realization of Poisson noise was different for each trial, but with a rate modulation that was repeated across trials.

### Spike Train Analysis

After learning, the analysis of certain network properties was contingent upon whether or not neurons participated in an attractor state. We adopted previously used criteria for detecting active attractor states in these types of networks [151]:

$$r_{a}(t)>c\cdot \sigma (t)>\underset{k\in \{0,\ldots ,N_{MC}\}\backslash a}{\text{max}}r_{k}(t).$$ (10)

The condition holds that the instantaneous firing rate of the most active attractor with index *a*, *r*_*a*_(*t*), is greater than the instantaneous standard deviation of the average firing rates of all attractors *σ*(*t*), which itself is greater than the instantaneous firing rate of the second most active attractor with index *k*, *r*_*k*_(*t*). In order to be counted as an active attractor, we required that index *a* did not change for at least 25 ms in order to filter out spurious activations. We found that reasonable variations of this cutoff value along with the numerical constant *c* = 1 that scaled *σ*(*t*) did not qualitatively impact our results. Detecting attractors in this way allowed at most one attractor to be active at a time, which reflected the propensity of the network to either be dominated by the activity of a single attractor or be in a ground state where there was competition amongst several slightly active populations. Furthermore it was advantageous due to the fact that *r*_*a*_(*t*) was a temporally local measure meaning that large, noisy activations due to complex feedback interactions did not bias detection at other points in time [151].

This information was in turn used to assess mesoscopic aspects of network function during recall such as attractor dwell times, sequence speeds, distribution of attractor transitions, and recall performances. The period of time in which the most active attractor with index *a* remained stable was recorded as the dwell time of that active attractor, *t*_*dwell*_. Sequence speeds in attractors per second could then be calculated by inverting the average *t*_*dwell*_, which was referred to as *μ*_*dwell*_, for all attractors replayed during recall:

$$\text{speed}=\frac{1}{\mu_{dwell}}.$$ (11)

Trained speed refers to the number of attractors per second that were sequentially stimulated at controlled durations during training, whereas recalled speed is the number of attractors per second replayed as a result of the learning that occurred during training. They were used to compute the compression factor according to:

$$\text{compression factor}=\frac{\text{recalled speed}}{\text{trained speed}}.$$ (12)

The conditional response probability (CRP) [152,153] curve was examined in order to evaluate the distribution of attractor transitions during recall. It represented the fraction of times a recalled attractor was followed by another attractor with a certain lag, where the index of the recalled attractor was relative to the index of its predecessor during training. Positive lags denoted forward transitions and negative lags denoted backward transitions. Time lags in the range -4…5 were adopted for full wrap around coverage of attractor position in the temporal domain, although self-transitions, i.e. time lag 0, were effectively unattainable due to residual adaptation and short-term depression. CRP values approaching 1 implied greater certainty in a transition. Since there were 10 patterns in this network, a CRP of 0.1 represented chance level for a transition.

Sequence recall fidelity was quantified by comparing the index order of detected attractors during recall with that of the trained stimulus template using the Levenshtein distance (i.e. edit distance) [154]. The difference between string representations of the sequentially trained attractor order *T* and the recalled attractor order *R* was calculated as the minimum number of single character edits needed to convert between *T* and *R*. The method penalized each required insertion, deletion, and substitution:

$$D_{RT}(u,v)=\{\begin{array}{ll} \;0 & \;;u=v=0\\ \;u & \;;v=0,u<0\\ \;v & \;;u=0,v<0\\ \text{min}\{\begin{array}{c} D_{RT}(u-1,v)+1\;\\ D_{RT}(u,v-1)+1\;\\ D_{RT}(u-1,v-1)+[R_{u}\neq T_{v}] \end{array} & \;;v>0,u>0. \end{array}$$ (13)

Here, *u* and *v* denote matrix indices, (…) the matrix element at the given indices and […] the indicator function that returned 0 when *R*_*u*_ = *T*_*v*_ and 1 otherwise. The reported edit distance *D*_*L*_ was the resulting lower right element of the matrix *D*_*RT*_. Thus *D*_*L*_ = 0 only when the recalled sequence episode was an exact match to the trained stimulus template.

The trained stimulus template *T* comprised only a single sequence that was repeated with different Poisson noise added each time. But in cases where attractors were cyclically activated by background activity and detected over the entire recall period, sequence replay success could be mixed, leading to varying individual recall patterns *R*. Sometimes the sequence was able to fully complete, but other times recall was truncated. Thus a single *D*_*L*_ value could not accurately summarize this heterogeneity.

For specific situations in which sequences degraded at the margins of compatible parameter sets, we observed that the first and last patterns tended to replay even when the patterns in the middle did not. These serial position effects, or the tendency for primacy and recency during sequence recall [1,153], meant that we could split the entirety of all concatenated yet separate recalled sequences based on delimiters corresponding to the first or last element indices. Using this criteria, we calculated the mean edit distance $\bar{D_{L}}$ by averaging *D*_*L*_ between the stimulus template and each individual recalled sequence. The maximum acceptable tolerance was set such that sequences with edit distances $\bar{D_{L}}$ > 5 were too degenerated to be considered successfully recalled. The expanded parameter sets of Figs 8–10 were guided by this criteria. By eliminating this criteria it was possible to demonstrate that sequence recall quality degraded for fast training speeds and in some cases for slow training speeds as well (S10 Fig).

## Supporting Information

S1 FigIncreasingly precise estimation of relative neuronal activity based on *τ*_*p*_.The standard deviation of $I_{\beta_{j}}$ measured for 50 randomly selected neurons per attractor after 100 training epochs shows that the terminal variability of $I_{\beta_{j}}$ decreases as a function of ***τ***_*p*_.(TIF)

S2 FigEvolution of local synaptic variables for learning sequential attractor states.(A) Development of average $g_{ij}^{AMPA}$ during training that project from neurons belonging to the stimulated first attractor, with color denoting target postsynaptic neuron attactor membership. In contrast to Fig 3E, the *g*_*ij*_ trajectories towards the two temporally adjacent (i.e. surrounding) attractors diverge from the other negative *g*_*ij*_. (B) Development of *P*_*ij*_ traces color corresponding to A and averaged over all NMDA synapses, which illustrates the underlying difference responsible for tilting the symmetry of $g_{ij}^{NMDA}$ when the IPI = 0. Although not shown here, the remaining synaptic traces *P*_*i*_ and *P*_*j*_ all converge to 0.1 for both the IPI = 0 and IPI = 2000 networks since there are 10 attractors and neurons fire at *f*_*max*_ 10% of the time, as evident from Figs 3A and 4A. (C) Average $g_{ij}^{NMDA}$ after training that depicts an asymmetrical and reversed terminal weight profile. Contrast with Fig 4C.(TIF)

S3 Fig Limited temporal extent of sequence replay illustrated by the gradual shift from reliably sequential to randomly wandering recalled attractor states with increasing IPI. This cross-section of the CRP curves depicts the lag 1 point from Fig 5B. Error bars reflect standard deviations.
(TIF)

S4 FigPrevailing ‘U’ shape of $\bar{D_{L}}$ in response to individual parameter changes illustrates the functional importance of different plasticity mechanisms.Systematic shutting off of **(A)** intrinsic excitability, **(B)** AMPA connections (100% = baseline), **(C)** adaptation, **(D)** NMDA connections (100% = baseline) and **(E)** short term plasticity each degrade the ability of the network to replay sequences. Error bars reflect standard deviations and red dotted lines denote $\bar{D_{L}}$ = 10 (i.e. maximal difference between trained and recalled sequences, see Eq 13) during 1 minute of replay.(TIF)

S5 FigGradually introducing random training patterns makes replay more random when the IPI = 0 ms.**(A)** Average $g_{ij}^{NMDA}$ after training as in Fig 4C (reproduced here by the 0.0 line) except now depicting terminal weight profiles for many differently trained networks with *P*(switch) varying between 0.0 and 1.0. **(B)** CRP curves calculated for networks with representative *P*(switch) = 0.0, 0.25, 0.5, 0.75 and 1.0 ms after 1 minute of recall, with colors corresponding to (A). Increasing *P*(switch) flattened the CRP curve, promoting attractor transition distribution evenness. Error bars reflect standard deviations.(TIF)

S6 Fig Variability of spike firing for attractor sequences. **(A)** The histogram of ISIs (10 ms binsize) of all excitatory cells during recall is bimodal, with lower ISIs reflecting periods in which neurons fire as part of an attractor, and higher ISIs reflecting the periodicity of attractor repeats. The gray dotted line indicates the average dwell time for the network, which demarcated the two distributions. **(B)** Highly variable spike trains for all excitatory pyramidal and inhibitory basket cells as portrayed by the mean local coefficient of variation (*CV*_*2*_, see Eq 4 in S1 Appendix). Error bars mark the standard deviation.
(TIF)

S7 FigChanging the number of training epochs demonstrates fast and stable learning.Average $g_{ij}^{NMDA}$ after training as in Fig 4C (reproduced here by the 50 epoch line). The distribution of weights converges to consistent set of values after only 10 epochs, and remains relatively stable up to 100 repeated epochs.(TIF)

S8 FigSequence disambiguation with one overlapping subsequence element.**(A)** Schematic of the training pattern as in Fig 11D demonstrating the problem of sequence disambiguation with one overlapping subsequence element. **(B)** Terminal average $g_{ij}^{NMDA}$ matrix resulting from (A), white and black Roman numerals as in Fig 11E. **(C)** Two separate cues (red and blue stars) presented 8 seconds apart each resonate through their corresponding subnetworks.(TIF)

S9 Fig CRP curve for the non-Markovian state transitioning network from Fig 11D–11F. CRP curve from dynamics of Fig 11D–11F retains a preference for lag 1 transitions (compare with Fig 5B and S5B Fig).
(TIF)

S10 FigSequence replay quality assessed using expanded edit distance tolerance levels.Average edit distances without the restriction that $\bar{D_{L}}$ ≤ 5 for recalled speeds of Fig 9 indicate a degradation of replay quality at the fastest and sometimes slowest speeds, which is quantified by modulating **(A)** the rate of background excitation, **(B)** the AMPA/NMDA ratio, **(C)** the magnitude of neural adaptation, **(D)** the magnitude of short-term depression, **(E)** the time constant of neural adaptation, and **(F)** the time constant of short-term depression.(TIF)

S1 Appendix Basis for BCPNN plasticity and model details. (DOCX)
