# Supplementary figures and images for: Spike-Based Bayesian-Hebbian Learning of Temporal Sequences
Source: PLoS Comput Biol. 2016 May 23;12(5):e1004954. doi: 10.1371/journal.pcbi.1004954 (PMC4877102; doi:10.1371/journal.pcbi.1004954)

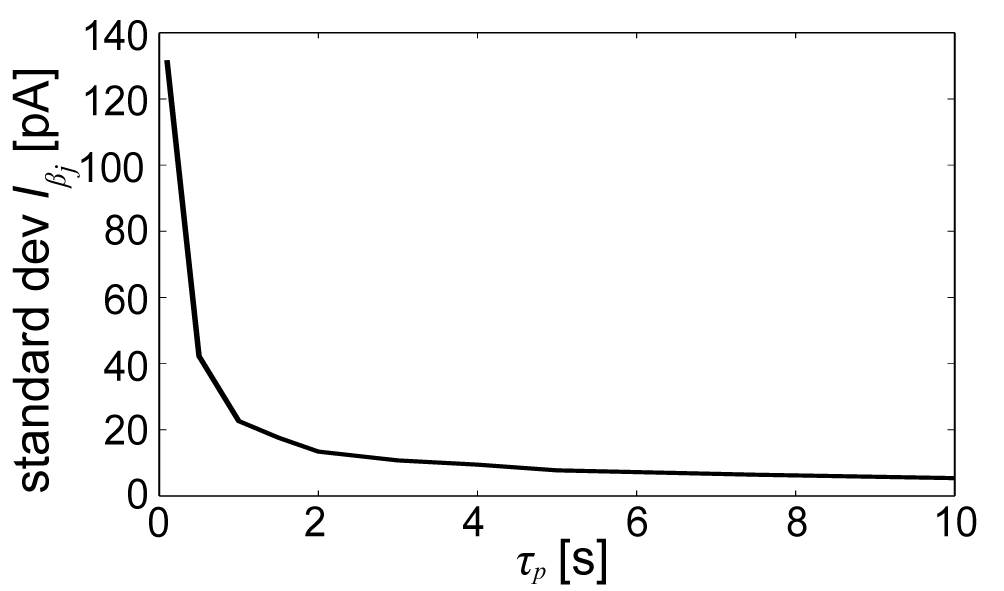

Supplement: S1 Fig — The standard deviation of Iβj measured for 50 randomly selected neurons per attractor after 100 training epochs shows that the terminal variability of Iβj decreases as a function of τp. (TIF) [file pcbi.1004954.s001.tif]

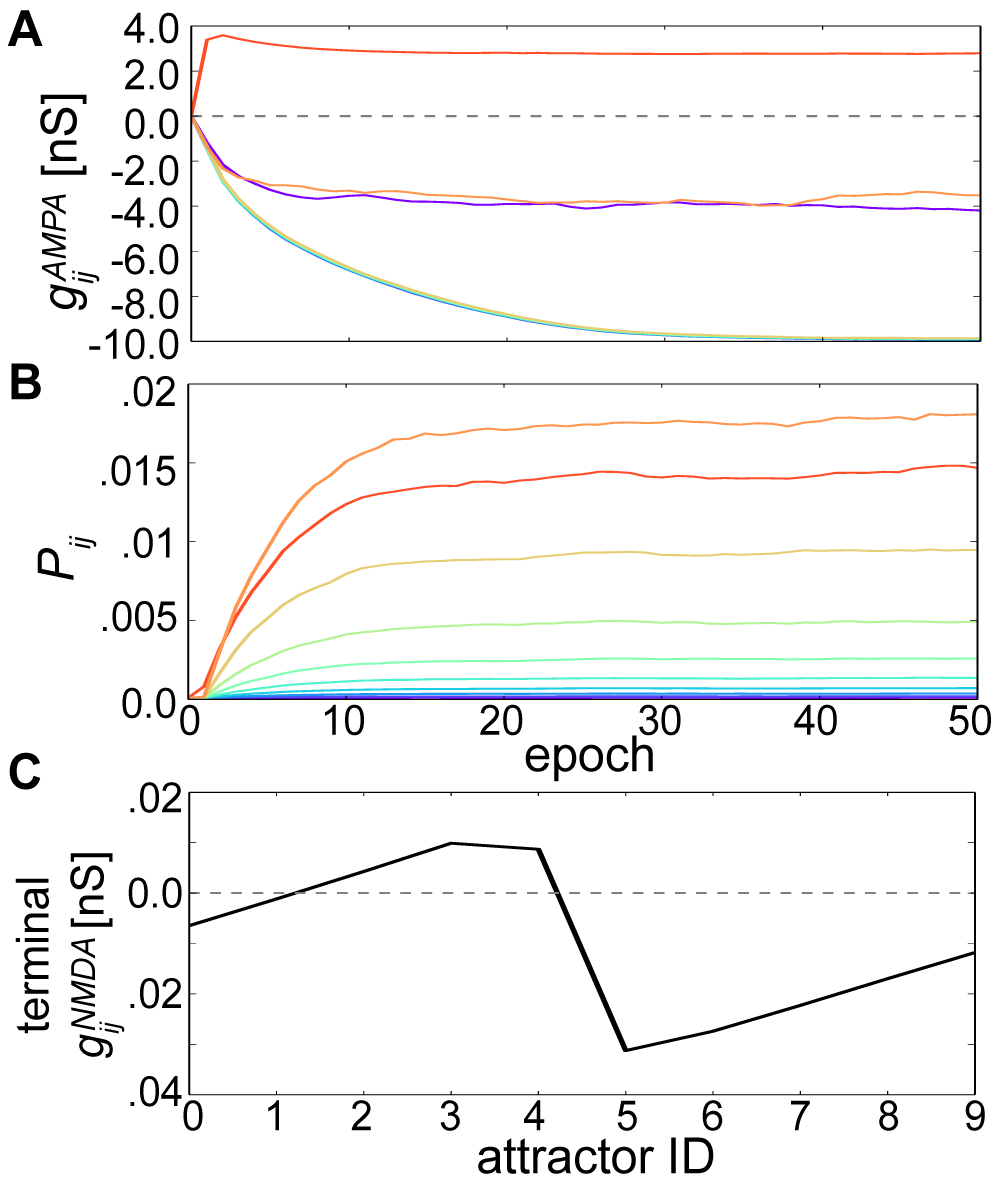

Supplement: S2 Fig — (A) Development of average gijAMPA during training that project from neurons belonging to the stimulated first attractor, with color denoting target postsynaptic neuron attactor membership. In contrast to Fig 3E, the gij trajectories towards the two temporally adjacent (i.e. surrounding) attractors diverge from the other negative gij. (B) Development of Pij traces color corresponding to A and averaged over all NMDA synapses, which illustrates the underlying difference responsible for tilting the symmetry of gijNMDA when the IPI = 0. Although not shown here, the remaining synaptic traces Pi and Pj all converge to 0.1 for both the IPI = 0 and IPI = 2000 networks since there are 10 attractors and neurons fire at fmax 10% of the time, as evident from Figs 3A and 4A. (C) Average gijNMDA after training that depicts an asymmetrical and reversed terminal weight profile. Contrast with Fig 4C. (TIF) [file pcbi.1004954.s002.tif]

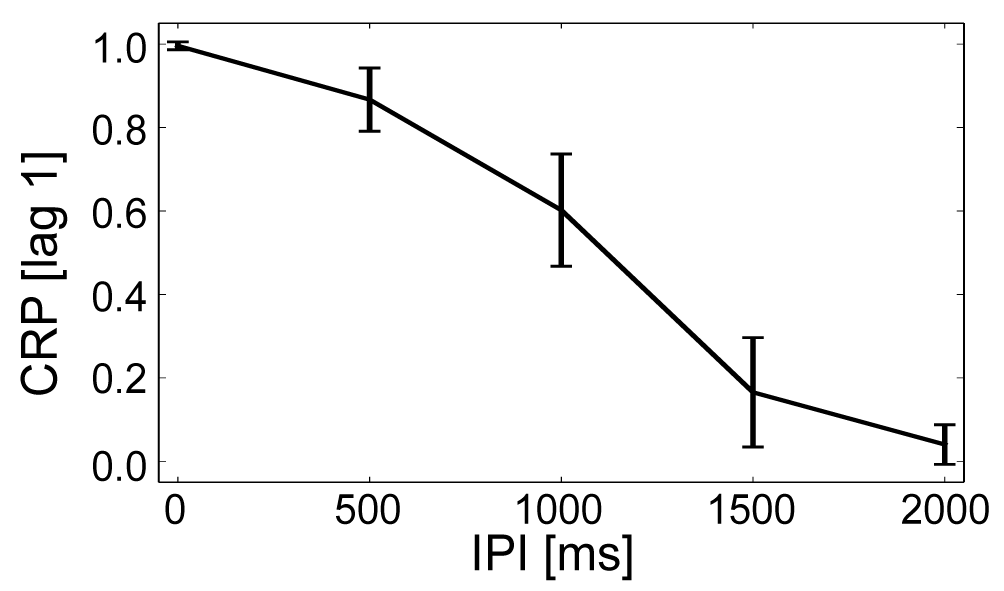

Supplement: S3 Fig — This cross-section of the CRP curves depicts the lag 1 point from Fig 5B. Error bars reflect standard deviations. (TIF) [file pcbi.1004954.s003.tif]

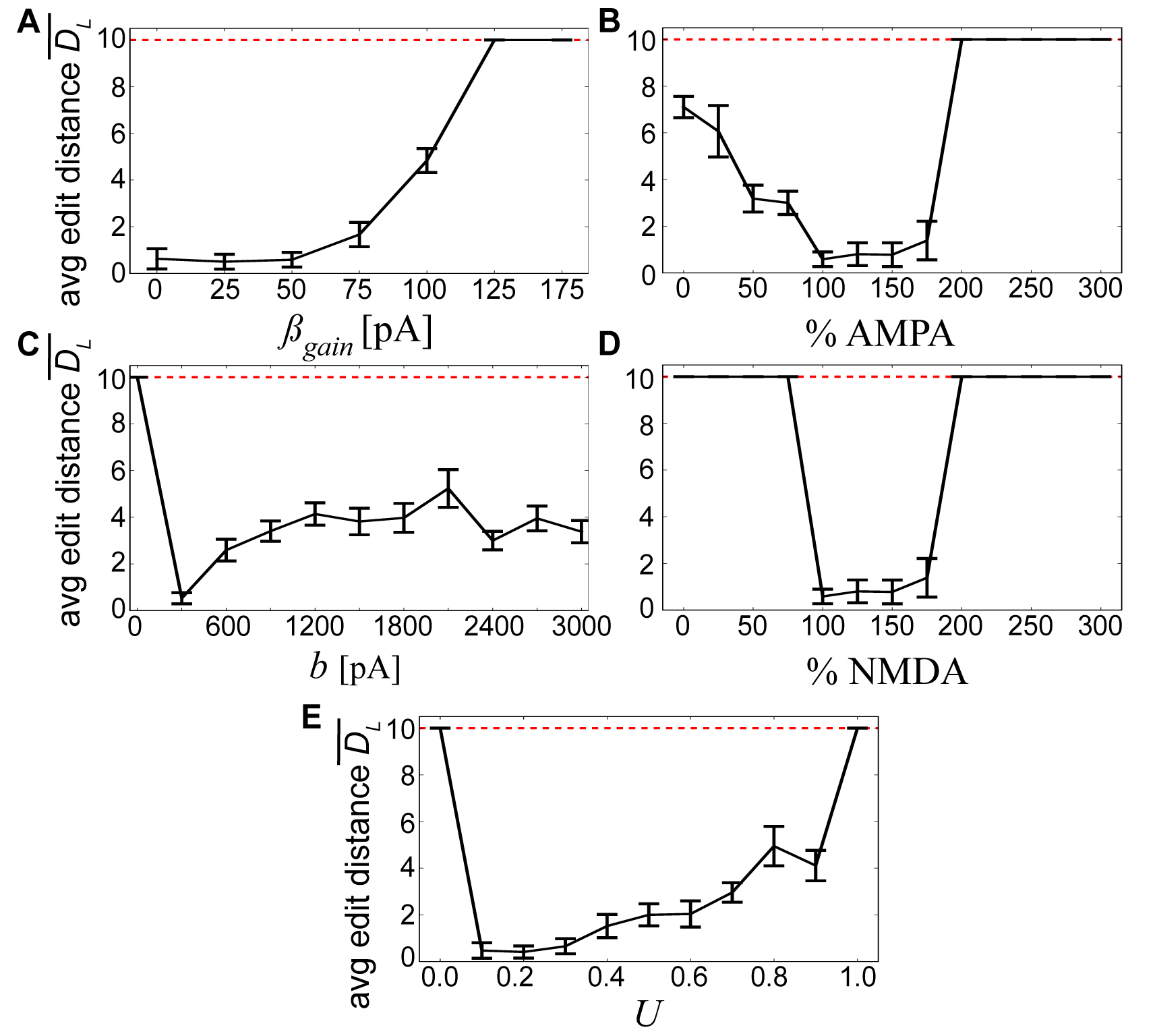

Supplement: S4 Fig — Systematic shutting off of (A) intrinsic excitability, (B) AMPA connections (100% = baseline), (C) adaptation, (D) NMDA connections (100% = baseline) and (E) short term plasticity each degrade the ability of the network to replay sequences. Error bars reflect standard deviations and red dotted lines denote DL¯ = 10 (i.e. maximal difference between trained and recalled sequences, see Eq 13) during 1 minute of replay. (TIF) [file pcbi.1004954.s004.tif]

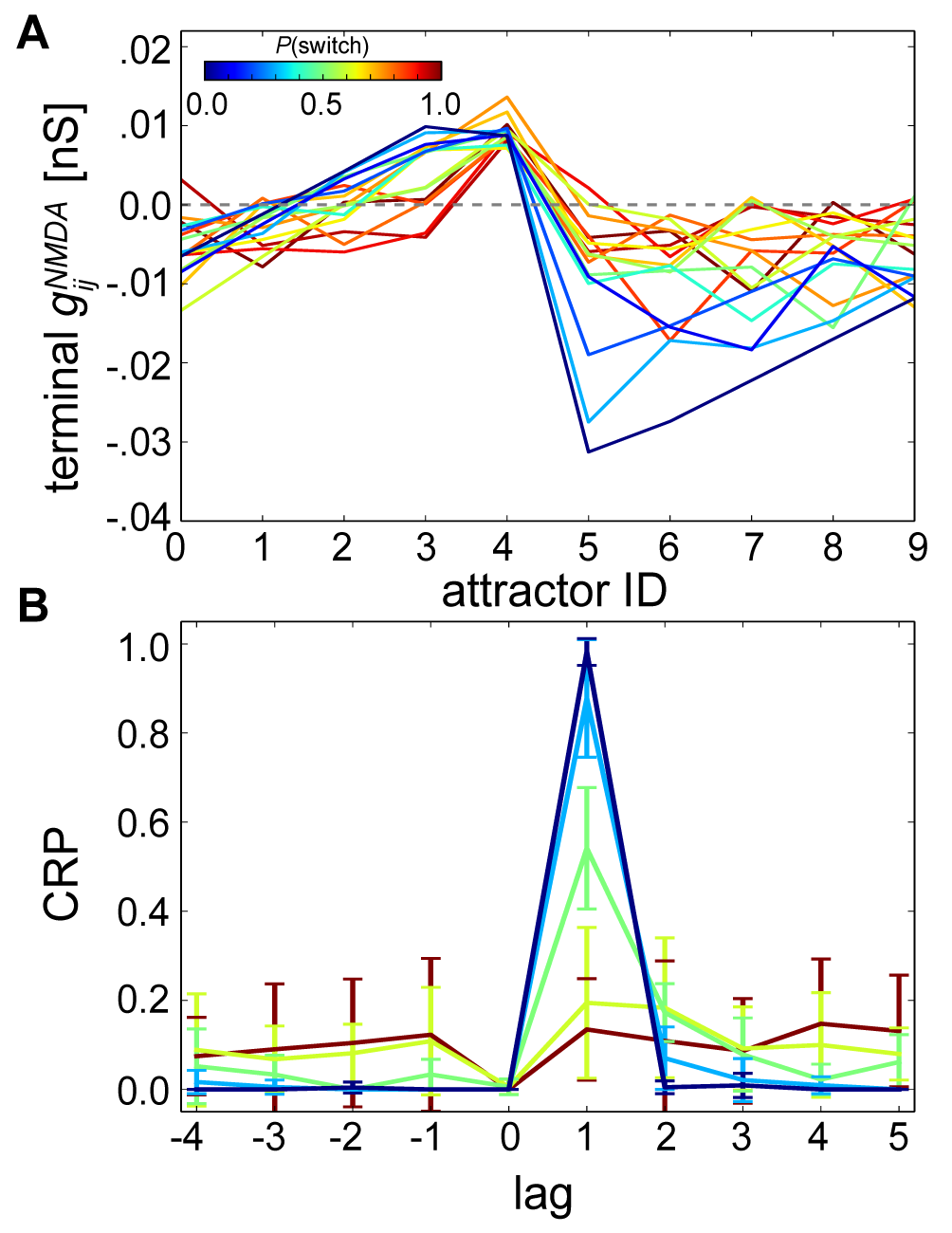

Supplement: S5 Fig — (A) Average gijNMDA after training as in Fig 4C (reproduced here by the 0.0 line) except now depicting terminal weight profiles for many differently trained networks with P(switch) varying between 0.0 and 1.0. (B) CRP curves calculated for networks with representative P(switch) = 0.0, 0.25, 0.5, 0.75 and 1.0 ms after 1 minute of recall, with colors corresponding to (A). Increasing P(switch) flattened the CRP curve, promoting attractor transition distribution evenness. Error bars reflect standard deviations. (TIF) [file pcbi.1004954.s005.tif]

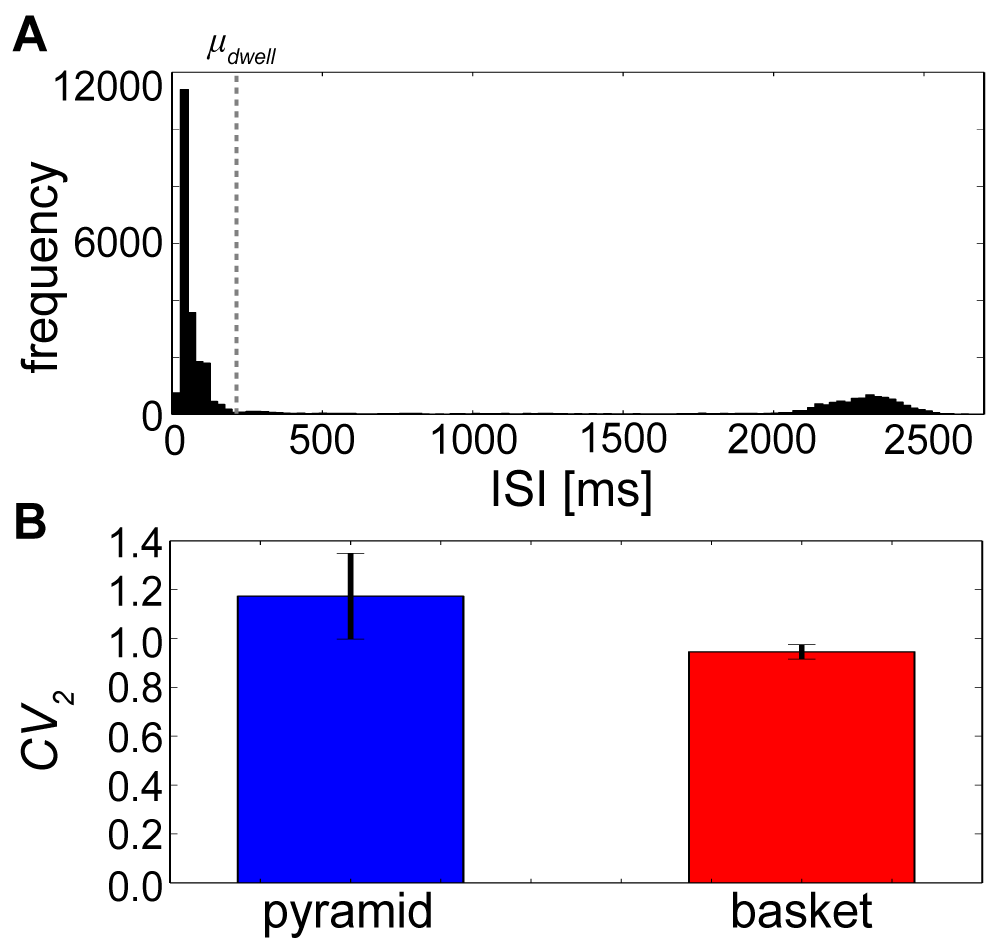

Supplement: S6 Fig — (A) The histogram of ISIs (10 ms binsize) of all excitatory cells during recall is bimodal, with lower ISIs reflecting periods in which neurons fire as part of an attractor, and higher ISIs reflecting the periodicity of attractor repeats. The gray dotted line indicates the average dwell time for the network, which demarcated the two distributions. (B) Highly variable spike trains for all excitatory pyramidal and inhibitory basket cells as portrayed by the mean local coefficient of variation (CV2, see Eq 4 in S1 Appendix). Error bars mark the standard deviation. (TIF) [file pcbi.1004954.s006.tif]

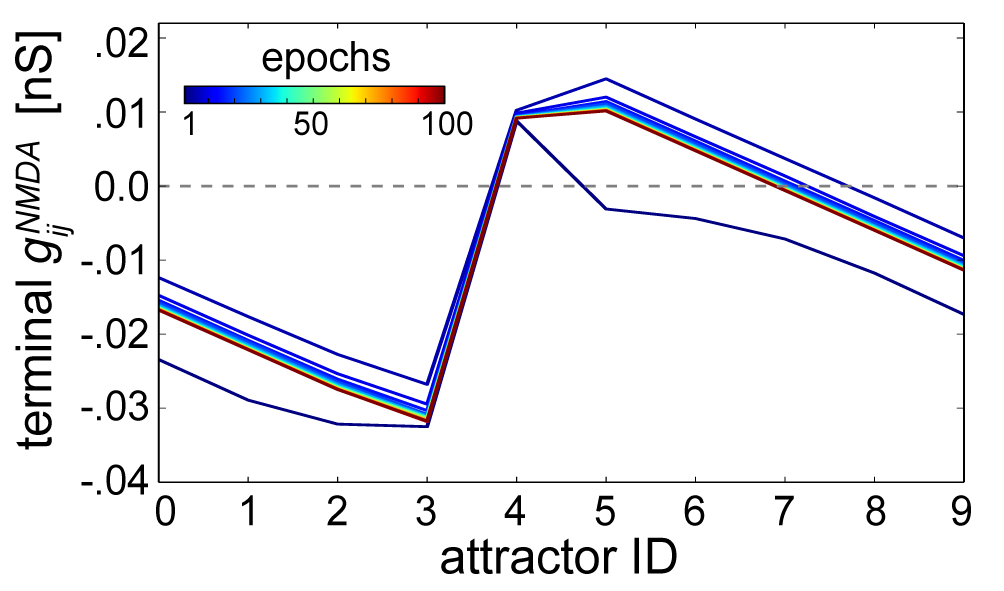

Supplement: S7 Fig — Average gijNMDA after training as in Fig 4C (reproduced here by the 50 epoch line). The distribution of weights converges to consistent set of values after only 10 epochs, and remains relatively stable up to 100 repeated epochs. (TIF) [file pcbi.1004954.s007.tif]

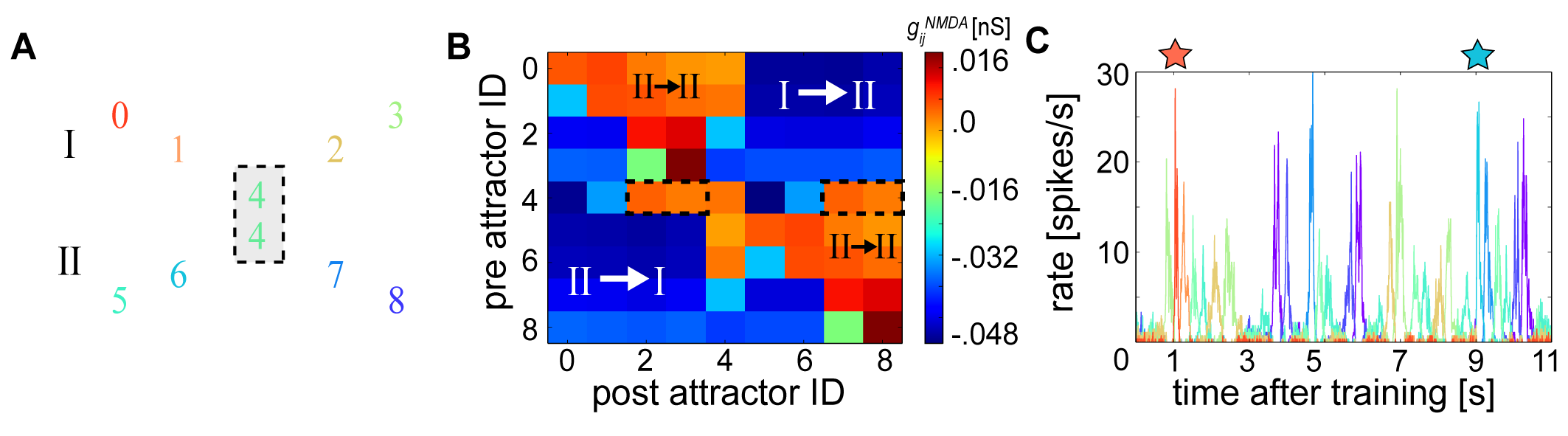

Supplement: S8 Fig — (A) Schematic of the training pattern as in Fig 11D demonstrating the problem of sequence disambiguation with one overlapping subsequence element. (B) Terminal average gijNMDA matrix resulting from (A), white and black Roman numerals as in Fig 11E. (C) Two separate cues (red and blue stars) presented 8 seconds apart each resonate through their corresponding subnetworks. (TIF) [file pcbi.1004954.s008.tif]

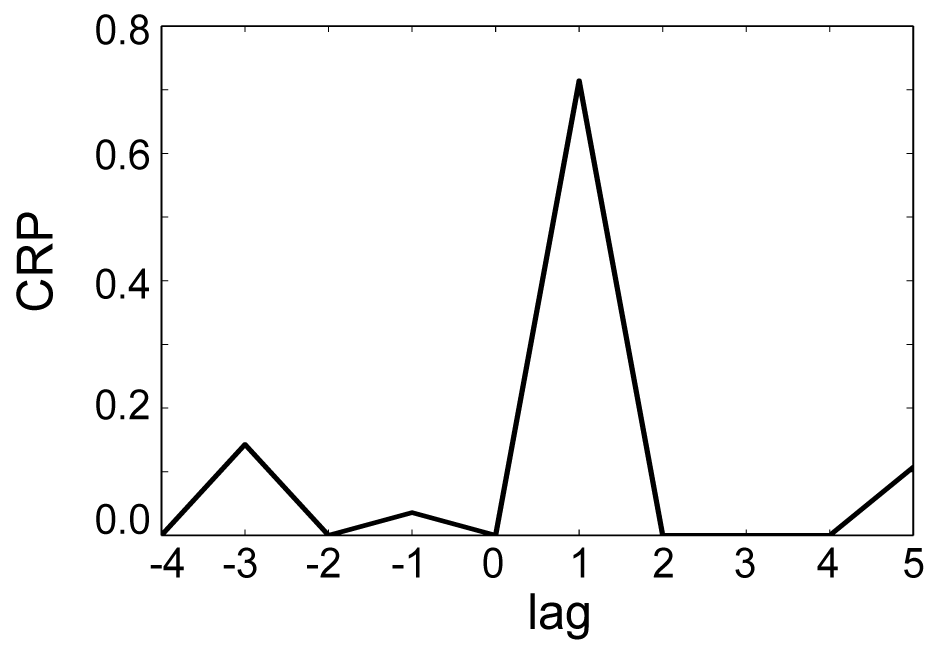

Supplement: S9 Fig — CRP curve from dynamics of Fig 11D–11F retains a preference for lag 1 transitions (compare with Fig 5B and S5B Fig). (TIF) [file pcbi.1004954.s009.tif]

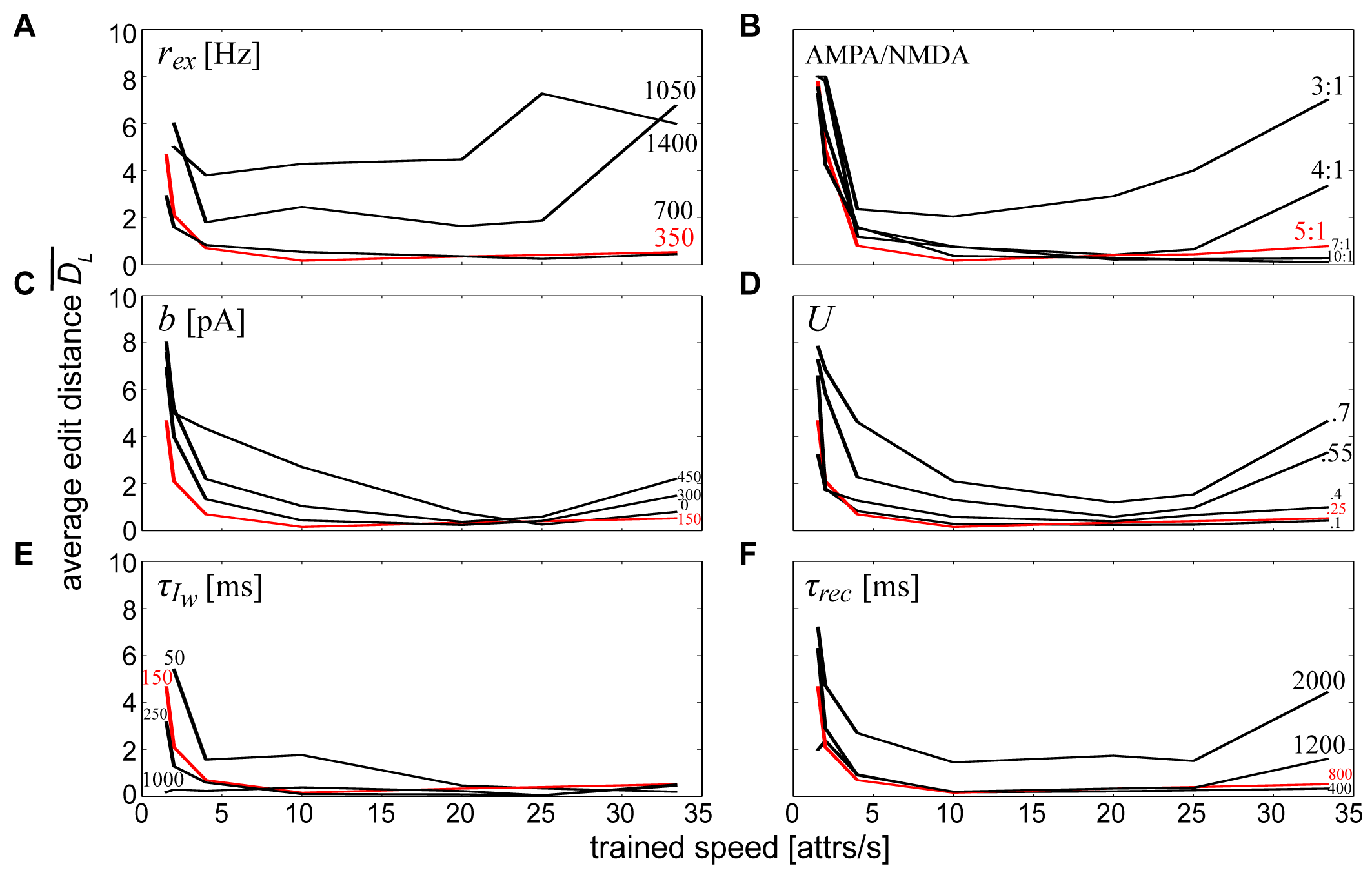

Supplement: S10 Fig — Average edit distances without the restriction that DL¯ ≤ 5 for recalled speeds of Fig 9 indicate a degradation of replay quality at the fastest and sometimes slowest speeds, which is quantified by modulating (A) the rate of background excitation, (B) the AMPA/NMDA ratio, (C) the magnitude of neural adaptation, (D) the magnitude of short-term depression, (E) the time constant of neural adaptation, and (F) the time constant of short-term depression. (TIF) [file pcbi.1004954.s010.tif]
