# Supplementary material for: Spike-Based Bayesian-Hebbian Learning of Temporal Sequences
Source: PLoS Comput Biol. 2016 May 23;12(5):e1004954. doi: 10.1371/journal.pcbi.1004954 (PMC4877102; doi:10.1371/journal.pcbi.1004954)
Supplement: S1 Appendix — (DOCX) [file pcbi.1004954.s011.docx]

***S1 Appendix***

**Basis for BCPNN Plasticity**

To motivate the probabilistic approximations carried out by plastic neural and synaptic components with dynamics according to Eq 4 in the main text, we digress into an abstract network configuration. It was previously shown that the *P* traces of Eq 3 in the main text could be combined to implement Bayesian inference where the connections and minicolumn activations have a statistical interpretation [35,141]. In this framework, observed firing rates collected from *N_MC_N_HC_* presynaptic minicolumns *x_1…NMCNHC_* inform the firing probabilities of neurons in the postsynaptic minicolumn *y_j_*. Given *x_h_* can be a discrete coded or interval coded continuous variable *x_hi_*, the network can be modularized such that all minicolumns (total of *N_MC_N_HC_*) are distributed into *N_HC_* hypercolumns (see Fig 1A):

 (1)

The activity of a minicolumn *i* of hypercolumn *h*, *π_xhi_* ∈ [*ε*, 1], corresponds to the mean firing rates of its neurons in probability space. In this Bayesian formulation of two discrete random variables *x* and *y*, the prior activation of the postsynaptic minicolumn *y_j_*, *P*(*y_j_*), combined with the activation of presynaptic minicolumns representing the likelihood, gives the posterior activation log *P*(*y_j_|x_1…NMCNHC_*) of *y_j_*.

A support value *s_j_ = β_yj_ +^^π_xi_w_xiyj_* can then be calculated, and the bias *β_yj_* and weight *w_xiyj_* terms identified from Eq 1:

$\beta_{y_{j}}=\log P(y_{j}), w_{x_{i}y_{j}}=\log\frac{P(x_{i}|y_{j})}{P(x_{i})}=\log\frac{P(x_{i},y_{j})}{P(x_{i})P(y_{j})}.$ (2)

We map the estimated *P* traces (Eq 3 in the main text) onto the derived terms of Eq 2 by noting the equivalence between *P_i_* and *P*(*x_i_*), *P_j_* and *P*(*y_j_*), and *P_ij_* and *P*(*x_i_, y_j_*). Posterior probabilities *P*(*y_j_|x_1…NMCNHC_*) approximated by minicolumn activity *π_yj_* are recovered from support values using an exponential transfer function:

$\pi_{y_{j}}=\frac{e^{s_{j}}}{\sum_{k=1}^{N_{MC}} e^{s_{k}}}.$ (3)

Here, *e^sj^* represents the activity of minicolumn *y_j_*. Normalization of activity approximately corresponding to Eq 3 is assumed to take place within each hypercolumn due to basket cell feedback inhibition among its minicolumns [1]. This normalization is not exact, but holds in the limiting WTA state.

**Model Details**

We used the mean local coefficient of variation (*CV_2_*) to measure the spike train ISI variability [2]:

$CV_{2}=\frac{1}{n-1}\sum_{i=1}^{n-1} \frac{2\left|ISI_{i}-ISI_{i+1}\right|}{ISI_{i}+ISI_{i+1}}.$ (4)

Thus for each spike train, the degree of variability of ISIs was evaluated using their *n* emitted spikes. Simulation parameters are interleaved below following [3].

| A | Model Summary |
| --- | --- |
| Neuron model | Adaptive exponential IAF |
| Synapse model | Conductance-based with exponentially shaped PSCs  Plastic spike-based BCPNN synapses |
| Channel model | CAN channel |
| Input model | Fixed-rate Poisson spike trains |
| Measured quantities | Spike activity, currents, biases, voltages, synaptic strengths |

| B | Neuron Model |
| --- | --- |
| AdEx IAF dynamics | - Subthreshold membrane potential *V_m_*:     Spiking: If *V_m_* ≥ *V_t_* a spike is generated and *V_m_* resets to *V_r_*   - Spike-triggered adaptation current *I_w_*: $\tau_{I_{w}}\frac{dI_{w}}{dt}=-I_{w}$    Adaptation: When a spike is generated update *I_w_* → *I_w_* + *b*   - Current from presynaptic input on postsynaptic cell *j, I_totj_*(*t*): $I_{tot_{j}}(t)=\sum_{syn} \sum_{i} g_{ij}^{syn}(t)(V_{m_{j}}-E_{ij}^{syn})$   |
| Parameters | *C_m_*= 280 pF membrane capacitance  *g_L_* = 14 nS leak conductance  *E_L_* = -70 mV leak reversal potential  *Δ_T_* = 3 mV slope factor  *V_t_* = -55 mV membrane voltage threshold  *V_r_* = -70 mV membrane reset potential  *τ_Iw_* = 150 ms adaptation time constant  *syn* = AMPA, NMDA or GABA synapse indiactor $E_{ij}^{AMPA}$  = 0 mV AMPA reversal potential $E_{ij}^{GABA}$  = -70 mV GABA reversal potential $E_{ij}^{NMDA}$  = 0 mV NMDA reversal potential |

| C | Channel Model |
| --- | --- |
| Activity-dependent hyperpolarizing | - CAN/A-type K^+^ current of neuron *j,* *I_βj_* pA: $S_{j}(t)=\sum_{sp} \delta(t-t_{sp}^{j})$ $\tau_{p}\frac{dP_{j}}{dt}=\kappa(Z_{j}-P_{j})$ $I_{\beta_{j}}=\beta_{gain}\log(P_{j})$         |
| Parameters | $\tau_{z_{j}}^{NMDA}$ = $\tau_{z_{j}}^{AMPA}$ = 5 ms *Z* trace time constant  *τ_p_* = 5000 ms *P* trace time constant  *∆t* = 1 ms spike duration  *f_max_ =* 20 Hz, highest probability/firing frequency  *ε* = 0.01, lowest probability/firing frequency  *β_gain_* = 50 pA CAN current scaling factor  *κ* = 1.0 if relevant, 0.0 otherwise to freeze intrinsic plasticity |

| D | Synapse Model |
| --- | --- |
| exponentially-shaped PSC dynamics | Conductance $x_{ij}^{dep}(t)w_{ij}^{syn}$ increase from presynaptic spike: $g_{ij}^{syn}(t)=x_{ij}^{dep}(t)w_{ij}^{syn}e^{-\frac{t-t^{i}-t_{ij}}{\tau_{syn}}}H(t-t^{i}-t_{ij})$  |
| Distance-dependent axonal delay | Delay between neurons *i* and *j, t_ij_* ms: $\overline{t_{ij}}=\frac{d_{norm}\sqrt{(m_{i}-m_{j})^{2}+(n_{i}-n_{j})^{2}}}{V}+1 \text{ms,} t_{ij}\sim N(\overline{t_{ij}}, .1\overline{t_{ij}})$  |
| BCPNN synaptic strength | Synaptic strength between neurons *i* and *j, w_ij_* nS: $S_{i}(t)=\sum_{sp} \delta(t-t_{sp}^{i}) , S_{j}(t)=\sum_{sp} \delta(t-t_{sp}^{j})$ $\tau_{p}\frac{dP_{i}}{dt}=\kappa(Z_{i}-P_{i}), \tau_{p}\frac{dP_{j}}{dt}=\kappa(Z_{j}-P_{j}), \tau_{p}\frac{dP_{ij}}{dt}=\kappa(Z_{ij}-P_{ij})$ $w_{ij}^{syn}=w_{gain}^{syn}\log\frac{P_{ij}}{P_{i}P_{j}}$        |
| Short-term depression | Tsodyks-Markram model: $\frac{dx_{ij}^{dep}}{dt}=\frac{1-x_{ij}^{dep}}{\tau_{rec}}-Ux_{ij}^{dep}\sum_{sp} \delta(t-t_{sp}^{i}-t_{ij})$  |
| Parameters | $w_{gain}^{AMPA}$ *=* 6.02 nS $w_{gain}^{NMDA}$  *=* 1.22 nS  *τ_AMPA_ =* 5 ms AMPA time constant  *τ_NMDA_ =* 150 ms NMDA time constant  *τ_GABA_ =* 5 ms GABA time constant $t_{sp}^{i}$  = presynaptic spike arrival time  *t_ij_*  = distant dependent transmission delay  (*m,n*) ∈ {0,1,2} hypercolumn index  *d_norm_* = 0.75 mm relative distance  *V* = 0.2 mm/ms conduction velocity  *κ* = 1.0 if relevant, otherwise 0.0 to freeze synaptic plasticity $\tau_{z_{i}}^{AMPA}$  = 5 ms AMPA *Z_i_* trace time constant (=*τ_AMPA_*) $\tau_{z_{i}}^{NMDA}$  = 150 ms NMDA *Z_i_* trace time constant (=*τ_NMDA_*) $\tau_{z_{j}}^{AMPA}$  = 5 ms AMPA *Z_j_* trace time constant $\tau_{z_{j}}^{NMDA}$  = 5 ms NMDA *Z_j_* trace time constant  *τ_p_* = 5000 ms *P* trace time constant  *∆t* = 1 ms spike duration  *f_max_ =* 20 Hz, highest probability/firing frequency  *ε* = 0.01, lowest probability/firing frequency  *U* = 0.25 increment produced by spike  *τ_rec_ =* 800 ms recovery time constant |

| E | Input |
| --- | --- |
| Background | Poisson Generators $w_{ij}^{AMPA}$  = 5 nS per process  *r_ex_*  = 350 Hz firing rate, independent for each neuron |
| Stimuli | Poisson Generators $w_{ij}^{AMPA}$  = 5 nS per process  *r_ex_*  = 150 Hz firing rate, independent for each neuron  *t_stim_* = 100 ms stimulus duration |

| F Measurements |
| --- |
| *r_ex_* spike rate (spikes/second)  *g_ij_* synaptic weight between neurons *i* and *j* (nS)  *V_m_* membrane voltage of neuron *j* (mV)  *I_βj_* bias current magnitude (pA)  *I_AMPA,_ I_NMDA,_ I_GABA_* synaptic current magnitude (pA) |

**References**

[1] Carandini M, Heeger DJ, Movshon, JA (1997) Linearity and Normalization in Simple Cells of the Macaque Primary Visual Cortex. Journal of Neuroscience, 17, 8621-8644.

[2] Holt GR, Softky WR, Koch C, Douglas RJ (1996) Comparison of Discharge Variability In Vitro and In Vivo in Cat Visual Cortex Neurons. Journal of Neurophysiology, 75(5), 1806-1814.

[3] Nordlie E, Gewaltig M-O, Plesser HE (2009) Towards Reproducible Descriptions of Neuronal Network Models. PLoS Computational Biology 5: e1000456. doi:10.1371/journal.pcbi.1000456.
